# Three-dimensional interactive network: Mitochondrial-metabolic-calcium homeostasis driving Alzheimer’s disease

**DOI:** 10.1016/j.gendis.2025.101846

**Published:** 2025-09-10

**Authors:** Tingting Liu, Zongting Rong, Jingwen Li, Haojie Wu, Jianshe Wei

**Affiliations:** Institute for Brain Sciences Research, School of Life Sciences, Henan University, Kaifeng, Henan 475004, China

**Keywords:** Alzheimer’s disease, Calcium homeostasis imbalance, Metabolic dysregulation, Mitochondrial dysfunction, Molecular mechanisms

## Abstract

Alzheimer’s disease (AD) is a progressive neurodegenerative disorder characterized by cognitive decline and neuronal loss, with its pathogenesis tightly linked to a “pathological triad”—mitochondrial dysfunction, metabolic dysregulation, and calcium homeostasis imbalance. This triad forms a mutually reinforcing network that amplifies AD pathology, yet its precise causal relationships and clinical relevance remain incompletely understood. Here, we critically synthesize evidence from human studies, animal models, and *in vitro* systems to dissect how these dysfunctions interact *in vivo*: mitochondrial structural damage and bioenergetic failure (e.g., reduced cytochrome c oxidase activity) impair ATP production, triggering metabolic reprogramming (e.g., astrocytic Warburg-like glycolysis, lactate shuttle dysfunction) and disrupting calcium buffering via mitochondrial calcium uniporter (MCU) dysregulation. Conversely, metabolic stress (e.g., hyperglycemia-induced mitochondrial overload) and calcium overload (e.g., NMDA receptor hyperactivation) exacerbate mitochondrial damage through reactive oxygen species (ROS) bursts and mitochondrial permeability transition pore (mPTP) opening. These processes are further amplified by amyloid β-protein (Aβ) and tau pathology: Aβ oligomers directly inhibit mitochondrial respiration and activate calcium channels, while hyperphosphorylated tau disrupts mitochondrial trafficking and exacerbates metabolic enzyme dysfunction. We evaluate the clinical translatability of preclinical findings, highlighting inconsistencies (e.g., conflicting results of CoQ10 trials) and gaps (e.g., human-specific metabolic signatures). Finally, we propose a framework prioritizing multi-target therapies that disrupt the triad’s vicious cycle, emphasizing the need for biomarkers to stratify patients based on triad dysregulation patterns.

## Introduction

Alzheimer’s disease (AD) imposes a devastating burden on global health, with its pathogenesis long attributed to amyloid β-protein (Aβ) plaques and neurofibrillary tangles (NFTs). However, the failure of Aβ- and tau-targeted therapies underscores the need to revisit broader pathological networks.^1^^,^^2^ Mounting evidence points to a “pathological triad”—mitochondrial dysfunction, metabolic dysregulation, and calcium imbalance—as core drivers that interact with and amplify Aβ/tau toxicity.^3^, ^4^, ^5^ The “collapse” of mitochondria triggers a series of chain disasters: structural damage (such as crista rupture and loss of membrane integrity) leads to the breakdown of the respiratory chain, which in turn causes an ATP crisis and an outbreak of reactive oxygen species (ROS); autophagic failure (Aβ inhibits PINK1/Parkin) results in the accumulation of damaged mitochondria, turning cells into uncontrolled “garbage dumps”; imbalance in dynamics (dynamin-related protein 1 (DRP1)/optic atrophy 1 (OPA1)) causes mitochondrial fragmentation, ultimately interrupting the energy supply.^3^^,^^6^

Metabolic reprogramming further adds fuel to the fire; in terms of glucose metabolism, the hyperactivity of the “Warburg effect” in astrocytes hinders lactate shuttling, leading to neurons being “starved in droves” due to energy deficiency; In lipid metabolism, cholesterol deposition facilitates Aβ aggregation, and apolipoprotein E 4 (ApoE4) exacerbates lipotoxicity, resulting in the disruption of membrane fluidity; In amino acid metabolism, the inverted ratio of glutamate to GABA triggers excitotoxicity and inhibitory imbalance, overburdening neurons.^7^ The calcium “tsunami” delivers the final blow to neurons, and overactivation of N-methyl-d-aspartate (NMDA) receptors/inositol trisphosphate receptors (IP3Rs) triggers calcium sparks, which then form pathological calcium waves, leading to intracellular calcium overload; Once calcium-dependent proteases are activated, they promote the hyperphosphorylation of tau proteins, forming NFTs; The loss of control of the mitochondrial calcium channel mitochondrial calcium uniporter (MCU) causes the collapse of the membrane potential, ultimately triggering cell apoptosis.^8^^,^^9^ There is a “toxic synergy” between Aβ and tau. Aβ activates glycogen synthase kinase-3β (GSK-3β) to phosphorylate tau, and tau aggregation in turn promotes Aβ deposition, forming a lethal cycle; Signaling pathways undergo collective betrayal. The inhibition of phosphatidylinositol 3-kinase (PI3K)-protein kinase B (Akt), overactivation of mitogen-activated protein kinase (MAPK), and loss of control of GSK-3β constitute a “death triangle”; In terms of epigenetics, ApoE4 mutation, DNA hypomethylation, abnormal histone modifications, etc., accelerate the pathological process.

**Figure 1:**
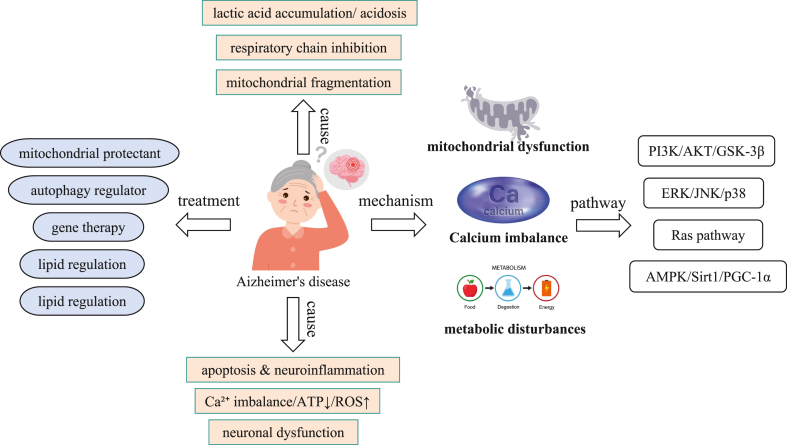
Associated factors, core mechanisms, and intervention methods of AD. In terms of pathogenic factors, it includes lactic acid accumulation/acidosis, respiratory chain inhibition, mitochondrial fragmentation, apoptosis and neuroinflammation, calcium imbalance/ATP reduction/ROS increase, and neuronal dysfunction, which can induce the disease. In terms of pathogenesis, mitochondrial dysfunction, calcium imbalance, and metabolic disorders are the key factors, and the disease process is mediated through signaling pathways such as PI3K/AKT/GSK-3β. Intervention strategies include therapeutic directions such as mitochondrial protectants, autophagy regulators, gene therapy, and lipid regulation.

Based on these findings, we propose a three-dimensional collaborative intervention strategy as a new dawn for treatment: First, construct mitochondrial armor, using Coenzyme Q10 as an antioxidant and autophagy activator to clear “garbage”^10^; Second, achieve metabolic rebalancing, using GLP-1 analogs to reverse glucose metabolism and statins to regulate lipids and block Aβ deposition^11^; Third, seal the calcium storm, using nimodipine to block calcium channels and quercetin to regulate calcium signals.^12^ By combining single-cell metabolomics with multi-omics integration, we will decode the spatiotemporal dynamics of this network and provide precise targets for early intervention. This is not only a mechanistic innovation but also a strategic turning point to end the impasse in AD treatment (Fig. 1).

## Normal “operation” of mitochondria, metabolism, and calcium homeostasis

### The “powerhouse” role of mitochondria

Mitochondria are double-membrane-bound organelles within cells, with their unique structure serving as the foundation for various critical physiological functions. The smooth outer membrane exhibits relatively high permeability, allowing small molecules and ions to pass freely. The inner membrane folds inward to form cristae, significantly increasing its surface area and providing attachment sites for key proteins such as respiratory chain complexes and ATP synthase.^13^ The mitochondrial matrix contains enzymes involved in metabolic pathways like the tricarboxylic acid (TCA) cycle and fatty acid oxidation, as well as mitochondrial DNA (mtDNA) and ribosomes, enabling independent gene expression and partial protein synthesis.^14^

As the cell’s “powerhouse”, mitochondria oxidize and break down nutrients such as carbohydrates, fats, and amino acids through oxidative phosphorylation (OXPHOS), converting the chemical energy stored in these substances into ATP to fuel various cellular activities.^15^ This process involves the TCA cycle and electron transport chain (ETC). During the TCA cycle, acetyl-CoA is fully oxidized, generating reducing equivalents such as NADH and FADH2. These equivalents transfer electrons to oxygen via the respiratory chain, while protons are pumped across the inner mitochondrial membrane, creating a transmembrane proton gradient that drives ATP synthase to produce ATP.^16^ Mitochondria also participate in biosynthesis; for example, multiple steps of heme synthesis occur within mitochondria, providing essential material foundations for cells.

### The “balance beam” of cellular metabolism

Cellular metabolism is a complex and orderly process involving the synthesis and breakdown of various substances, with glucose metabolism, lipid metabolism, and amino acid metabolism being the most critical pathways. Glucose metabolism primarily includes glycolysis, the TCA cycle, the pentose phosphate pathway, and gluconeogenesis. Glycolysis occurs in the cytoplasm, breaking down glucose into pyruvate while generating small amounts of ATP and NADH.^17^ Under aerobic conditions, pyruvate enters mitochondria to participate in the TCA cycle, where it is fully oxidized into carbon dioxide and water, releasing substantial energy. Under anaerobic conditions, pyruvate is reduced to lactate. The pentose phosphate pathway primarily produces NADPH and ribose phosphate. NADPH participates in intracellular reduction reactions, such as fatty acid and cholesterol synthesis, while ribose phosphate serves as a key precursor for nucleic acid synthesis.^18^ Gluconeogenesis synthesizes glucose from non-carbohydrate sources (e.g., lactate, glycerol, and glucogenic amino acids), playing a vital role in maintaining blood glucose stability.

Lipid metabolism encompasses fat synthesis, breakdown, and fatty acid β-oxidation. Fat synthesis involves combining glycerol and fatty acids into triglycerides for storage, primarily occurring in adipose tissue and the liver.^19^ Lipolysis, mediated by hormone-sensitive lipase, breaks down triglycerides into glycerol and fatty acids. Fatty acids undergo β-oxidation in mitochondria to generate acetyl-CoA, which enters the TCA cycle to release energy.^20^ Amino acid metabolism includes deamination, decarboxylation, and synthesis. Ammonia produced by deamination is converted to urea via the ornithine cycle in the liver for excretion, while α-keto acids enter glucose or lipid metabolism pathways to provide energy or synthesize other molecules.^21^

These metabolic pathways are interconnected and coordinated. For instance, pyruvate from glycolysis can be converted to acetyl-CoA for fat synthesis; glycerol from lipolysis can enter gluconeogenesis to form glucose; and α-keto acids from amino acid deamination can join glucose or lipid pathways.^22^ Cells maintain metabolic balance through precise regulatory mechanisms, including allosteric and covalent enzyme modifications, hormonal regulation, and gene expression control. Insulin promotes glucose uptake and utilization, inhibits gluconeogenesis, and enhances fat and protein synthesis, while glucagon elevates blood glucose, stimulates lipolysis, and activates gluconeogenesis.^23^

### The “precise regulation” of calcium homeostasis

Intracellular calcium homeostasis is essential for normal cellular physiology. At rest, the cytosolic calcium concentration ([Ca^2+^]i) is approximately 100 nM, while the extracellular levels are ∼1.2 mM, creating a steep concentration gradient.^24^ Cells maintain this gradient via plasma membrane calcium channels, pumps (e.g., Ca^2+^-ATPase), and intracellular calcium stores (ER and mitochondria). Plasma membrane channels include voltage-gated calcium channels (VGCCs), receptor-operated calcium channels (ROCCs, also termed ligand-gated calcium channels), and mechanosensitive channels.^25^ VGCCs are critical in excitable cells (e.g., neurons, cardiomyocytes), where membrane depolarization triggers Ca^2+^ influx, initiating processes like neurotransmitter release and muscle contraction. ROCCs open upon ligand binding (e.g., acetylcholine at neuromuscular junctions). Additionally, plasma membrane Ca^2+^-ATPase (PMCA) and sodium-calcium exchanger (NCX) extrude Ca^2+^ against the gradient using ATP hydrolysis or sodium electrochemical gradients, respectively.^26^ The ER, a major calcium store, releases Ca^2+^ via IP3Rs and ryanodine receptors (RyRs) upon stimulation, while sarco/ER Ca^2+^-ATPase (SERCA) pumps reuptake cytosolic Ca^2+^ to maintain high ER luminal Ca^2+^ levels.^27^ Mitochondria regulate calcium via MCU for uptake and NCX for efflux, balancing cytosolic and mitochondrial Ca^2+^ while modulating energy metabolism.^28^

As a key second messenger, Ca^2+^ regulates diverse physiological processes—muscle contraction, neurotransmitter release, proliferation, differentiation, and apoptosis—through dynamic concentration changes. Elevated [Ca^2+^]i promotes Ca^2+^ binding to calmodulin (CaM), forming Ca^2+^-CaM complexes that activate downstream effectors like Ca^2+^/CaM-dependent kinases (CaMKs), triggering specific responses.^29^ For example, Ca^2+^ binding to troponin initiates muscle contraction, while neuronal Ca^2+^ influx facilitates neurotransmitter release. Calcium signaling begins with extracellular Ca^2+^ influx or ER Ca^2+^ release (via IP3R/RyR), generating cytosolic Ca^2+^ transients. Calcium-induced calcium release (CICR) amplifies signals into propagating waves, activating pathways like CaMK and PKC to regulate metabolism and proliferation. Signal termination relies on Ca^2+^ clearance systems: PMCA and NCX extrude Ca^2+^, SERCA refills ER stores, and mitochondria buffer excess Ca^2+^ via MCU/NCX.^30^ Calcium-binding proteins (e.g., CaM, troponin) prevent overload by sequestering free Ca^2+^ and mediating signal transduction.^31^

**Figure 2:**
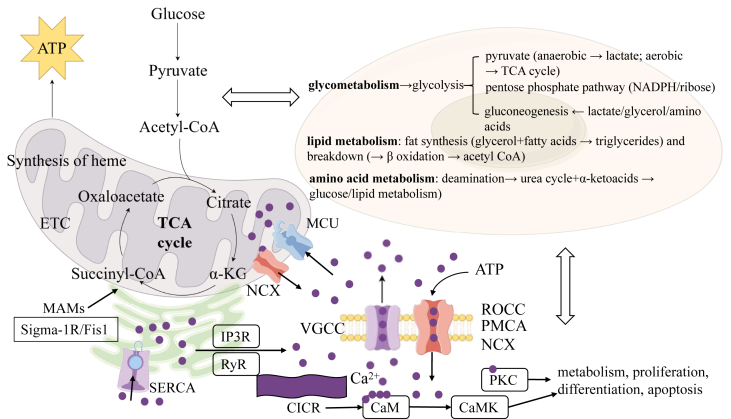
Mitochondria, as the “powerhouses” of the cell, drive OXPHOS through their double-membrane structure (permeable outer membrane and cristae-rich inner membrane) and matrix-residing enzyme systems (e.g., the TCA cycle and respiratory chain complexes), converting carbohydrates, lipids, and amino acids into ATP while participating in heme synthesis. Cellular metabolism, centered on the glucose, lipid, and amino acid pathways, maintains dynamic equilibrium via glycolysis, the TCA cycle, β-oxidation, and interconversion pathways (e.g., pyruvate→lipids, glycerol→gluconeogenesis), which is regulated by hormones such as insulin/glucagon. Calcium homeostasis relies on MAMs, which in AD exhibit MAM expansion (Sigma-1R/Fis1 ratio imbalance) and pathological transitions from calcium sparks to waves, causing calcium overload and protease-mediated cellular damage. Calcium signaling is triggered by VGCCs or ER release (IP3R/RyR), amplified via CICR, and terminated by PMCA, SERCA pumps, and calcium-binding proteins (e.g., calmodulin). Dysregulation of this system is linked to neurodegenerative and cardiovascular diseases. Together, these mechanisms reveal AD-associated dysregulation of the energy–metabolism–calcium signaling network, offering insights for targeted therapies.

Calcium homeostasis is finely tuned by genetic, oxidative, and inflammatory factors. Dysregulation leads to calcium overload, aberrant protease activation, and cellular damage, linking it to cardiovascular diseases, neurodegeneration, and cancer. Understanding these regulatory mechanisms holds significant therapeutic potential (Fig. 2).

## Aberrant “variations” in AD

### The “dilemma” of mitochondrial dysfunction

In the pathological progression of AD, mitochondrial dysfunction plays a pivotal role, severely impairing neuronal function and acting as a core driver of disease progression. This dysfunction encompasses multiple critical aspects, including structural damage, energy metabolism abnormalities, dynamic imbalance, and defective mitophagy, all of which are intricately interconnected to form a complex and lethal pathological network. The integrity of the mitochondrial structure is essential for maintaining normal function. Studies have revealed significant morphological alterations in mitochondria from AD brains, including swelling, crista fragmentation, reduced cristae density, and compromised outer/inner membrane integrity.^32^ These changes resemble cracks in a building’s load-bearing walls, disrupting the internal architecture and function of mitochondria, and directly interfering with the assembly and activity of key protein complexes such as the ETC and ATP synthase.^33^

Electron microscopy of AD brain tissue clearly demonstrates swollen mitochondria with fragmented cristae, which fail to efficiently perform energy metabolism, leading to neuronal energy deficits and subsequent pathological cascades.^33^ Further studies show mitochondrial degeneration in AD, characterized by hydropic changes, thinned matrix, and vacuolation.^33^ In AD rat neurons, mitochondria exhibit increased average volume, reduced surface area-to-volume ratio, and decreased numerical density, reflecting swelling and loss.^34^ Human AD neurons display mild mitochondrial swelling, structural disorganization, vacuolation, and crista disarray or disintegration in severe cases.^34^ Mitochondrial abnormalities in AD patients are well-documented: post-mortem brains show swollen mitochondria with fragmented cristae, reduced Complex I/IV activity, and mtDNA mutations correlated with the Braak stage.^35^ However, their temporal relationship with Aβ/tau remains debated. Longitudinal studies in AD mice suggest that mitochondrial respiration deficits precede Aβ deposition, while human data show Aβ plaques colocalize with dysfunctional mitochondria, supporting a feedforward loop.^36^

Mitochondrial energy metabolism abnormalities are a hallmark of AD, trapping neurons in an energy-deprived state. OXPHOS is severely suppressed in AD mitochondria, drastically reducing ATP production. This stems from ETC dysfunction, particularly the diminished activity of cytochrome C (Cyt C) oxidase, which disrupts electron transfer, proton gradient formation, and ATP synthesis.^37^ mtDNA mutations further exacerbate ETC dysfunction by encoding defective ETC proteins, reducing ATP output while increasing ROS production.^38^ AD brains exhibit higher mtDNA mutation rates compared to controls, which are correlated with metabolic deficits and neuronal damage.^39^ Impaired cerebral glucose uptake and utilization in AD contribute to mild cognitive impairment.^40^ Elevated cerebrospinal fluid lactate, reduced succinate/fumarate levels, and decreased activity of ETC complexes (I, II, III, and Cyt C) confirm mitochondrial oxidative damage.^41^ Age-related mtDNA instability in AD increases mutations, disrupting ETC function, ATP synthesis, and calcium homeostasis while increasing oxidative stress, protein/lipid oxidation, and mitochondrial permeability transition, accelerating neuronal aging and death.^42^

Mitochondrial dynamics—fusion, fission, transport, and positioning—are critical for functional and spatial homeostasis. In AD, fission/fusion imbalance arises from dysregulation of DRP1 and OPA1.^3^, ^4^, ^5^, ^6^ Overactivated DRP1 drives excessive mitochondrial fragmentation, while reduced or dysfunctional OPA1 impairs fusion.^3^, ^4^, ^5^, ^6^ This imbalance disrupts mitochondrial morphology, energy production, calcium buffering, and intracellular transport while elevating ROS.^3^, ^4^, ^5^, ^6^ AD-linked gene mutations (e.g., presenilin-1/2, PS1/PS2) exacerbate dynamic defects. For instance, PS1 mutations inhibit the mitochondrial fusion proteins Mfn1/Mfn2, promoting fragmentation.^43^

Mitophagy—the selective clearance of damaged mitochondria—is impaired in AD, akin to a malfunctioning cellular waste disposal system. Damaged mitochondria accumulate in AD neurons due to failed autophagosome–lysosome degradation. These dysfunctional mitochondria not only cease energy production but also generate excessive ROS, exacerbating cellular damage.^44^ AD-associated proteins (e.g., Aβ, tau) disrupt mitophagy by inhibiting key regulators like PTEN-induced kinase 1 (PINK1) and Parkin 45. For example, Aβ oligomers bind to PINK1, blocking its kinase activity and preventing Parkin recruitment to mitochondria, thereby halting mitophagy initiation.^46^

### The “vortex” of metabolic dysregulation

In AD, metabolic reprogramming exhibits unique spatiotemporal features. Astrocytes display hyperactive glycolysis, resembling the Warburg effect in cancer cells.^47^ This leads to excessive glucose uptake and lactate production via glycolysis in astrocytes, while neurons suffer from OXPHOS decompensation.^48^ Normally, neurons rely on OXPHOS for energy production. In AD, mitochondrial dysfunction and impaired substrate supply render neurons unable to efficiently generate sufficient energy via OXPHOS, resulting in an energy crisis.^49^ Lactate shuttle dysfunction represents another critical aspect of AD metabolic abnormalities. Epigenetic dysregulation of monocarboxylate transporters 1 and 4 (MCT1/4) disrupts lactate transport between astrocytes and neurons. The lactate shuttle is essential for maintaining brain energy homeostasis, and its impairment causes metabolic signaling decoupling, compromising neuronal energy supply and signaling,^50^ and lactate accumulation (from overproduction and reduced clearance), which disrupts pH and drives pathological post-translational modifications (PTMs). Protein lactylation (Kla), a lactate-dependent PTM, links metabolism to protein function and gene expression.^51^ It modulates histones (e.g., H3K18la) to up-regulate glycolytic genes, forming a feed-forward loop that exacerbates metabolic imbalance, neuroinflammation, and oxidative stress.

Lactylation of amyloid precursor protein (APP) promotes amyloidogenic processing, increasing neurotoxic Aβ42.^52^ This creates a cycle in which dysregulated glucose metabolism elevates lactate, enhancing APP cleavage and Aβ42 production; Aβ then worsens mitochondrial dysfunction and glucose uptake impairment. Lactylation also intersects with mitochondrial and calcium homeostasis, amplifying pathogenesis via ROS, disrupted calcium buffering, and perturbed signaling.^53^ Targeting glucose metabolism and lactylation (e.g., with inhibitors of lactate production/transport or lactylation regulatory enzymes, or combination strategies) shows therapeutic promise, highlighting the need for multi-target therapies addressing these interconnected pathways. Single-cell metabolomics has revealed neuron subtype-specific metabolic vulnerabilities. Distinct neuronal populations exhibit varying sensitivities to metabolic disturbances in AD, with certain subtypes being more prone to energy dysregulation. This provides new insights into the selective neuronal damage mechanisms in AD.^54^

In AD, severe metabolic abnormalities in glucose, lipids, and amino acids intertwine within the brain, forming a self-reinforcing “vortex” that interacts bidirectionally with mitochondrial dysfunction to drive disease progression. The brain relies heavily on glucose for energy, which is normally converted to ATP via glycolysis and OXPHOS. However, AD brains exhibit early and critical glucose metabolism defects, including reduced uptake, impaired glycolysis, and suppressed OXPHOS, leading to energy deficits. Regions critical for cognition—such as the temporal, parietal, and frontal lobes—show significantly lower glucose metabolic rates than healthy individuals.^55^

Mitochondrial dysfunction exacerbates these defects by disrupting OXPHOS and ATP synthesis, which in turn results in the inhibition of glucose uptake and glycolysis. Impaired respiratory chain complexes reduce oxygen utilization, destabilizing energy balance and suppressing glucose transporter activity. AD pathological proteins like Aβ and tau further disrupt glucose metabolism: Aβ inhibits glucose transporters glucose transporter type 1 (GLUT1)/GLUT3, while hyperphosphorylated tau destabilizes microtubules, impairing transporter trafficking and localization.^56^^,^^57^ Compensatory activation of anaerobic glycolysis increases lactate production, disrupting the pH balance and damaging neurons. Positron emission tomography (PET) imaging reveals reduced glucose metabolism ratios in AD brains, particularly in the right parietal lobe, posterior temporoparietal regions, hippocampus, and posterior cingulate cortex.^58^ Lipids are essential for membrane structure and function, yet AD brains exhibit dysregulated synthesis, metabolism, and transport of cholesterol, triglycerides, and phospholipids.^59^ Elevated cholesterol, particularly around Aβ plaques, promotes Aβ aggregation and alters membrane fluidity, creating a vicious cycle that accelerates plaque formation.^60^ Lipid peroxidation generates ROS, damaging neurons and mitochondria.^61^ Mitochondrial dysfunction impairs fatty acid β-oxidation, leading to intracellular lipid accumulation. Defective mitochondrial fatty acid transporters reduce fatty acid import, while lipid peroxidation products (e.g., malondialdehyde) damage mitochondrial membranes and enzymes, further impairing respiratory chain activity and ATP synthesis.^62^ ApoE, particularly the ε4 isoform, exacerbates AD pathology by disrupting cholesterol efflux, promoting Aβ aggregation, and fostering NFT formation.^62^ Altered fatty acid metabolism destabilizes neuronal membranes, and toxic lipid peroxides amplify oxidative stress, accelerating neurodegeneration.^62^ Amino acid metabolism is critical for protein synthesis and signaling. AD brains show abnormal amino acid levels, altered transporter activity, and disrupted enzyme function. Glutamate levels rise, while γ-aminobutyric acid (GABA) levels decline.^63^ Excess glutamate causes excitotoxic neuronal damage via overactivation of NMDA receptors, while reduced GABA disrupts the inhibitory–excitatory balance, worsening neuronal injury.^64^ Mitochondria participate in amino acid metabolism (e.g., glutamate oxidation, urea cycle), and their dysfunction leads to toxic metabolite accumulation. For example, impaired glutamate-oxidizing enzymes increase glutamate levels, while ammonia (a byproduct of amino acid catabolism) inhibits respiratory chain complexes, reduces ATP synthesis, and increases ROS production.^65^

In terms of glucose metabolism, reduced cerebral glucose uptake (detected by PET) is an early biomarker, but the mechanistic connection between this reduction and mitochondria is highly complex.^66^ Although mitochondrial dysfunction impairs the process of OXPHOS, the increase of glycolysis in astrocytes (achieved through hypoxia-inducible factor (HIF-1α)) may play a compensatory role.^65^ Nevertheless, this compensatory mechanism leads to the accumulation of lactic acid and acidosis, which in turn exacerbates neuronal damage. At the level of lipid metabolism, carriers of ApoE4 have abnormal cholesterol transport, which promotes the aggregation of Aβ. Simultaneously, defects in mitochondrial β-oxidation also result in lipid peroxidation (such as the accumulation of 4-hydroxynonenal).^67^ However, the results of clinical trials on statins have been mixed, highlighting the necessity of distinguishing between targets for cholesterol synthesis and those for cholesterol transport. In amino acid metabolism, the imbalance between glutamate and GABA in AD patients not only reflects the abnormal function of mitochondrial glutamate dehydrogenase but also is related to calcium-dependent glutamate release. This directly links metabolic disorders and abnormal calcium regulation to excitotoxicity.

### The “crisis” of calcium homeostasis imbalance

Mitochondria-associated endoplasmic reticulum (ER) membranes (MAMs) play a critical role in calcium signaling and cellular homeostasis.^68^ In AD, MAMs exhibit abnormal expansion, characterized by an imbalance in the Sigma-1R/Fis1 ratio. Sigma-1R, a molecular chaperone protein, shows dysregulated interaction with the mitochondrial fission protein Fis1, impairing MAM structure and function.^69^ This pathological expansion disrupts calcium signaling between the ER and mitochondria, altering mitochondrial calcium uptake and buffering capacity.^70^

Spatiotemporal dynamics of intracellular calcium signaling undergo significant changes, transitioning from nanoscale calcium sparks to pathological calcium waves.^71^ Under normal conditions, calcium sparks are localized, transient signaling events. In AD, however, calcium homeostasis imbalance increases spark frequency and propagates their spread into calcium waves. These pathological waves induce intracellular calcium overload, activating calcium-dependent proteases and phosphatases that damage cellular structure and function.^72^ Optogenetic tools have provided novel insights into AD-associated calcium dysregulation. Studies have revealed that Aβ oligomers induce dendritic microdomain calcium oscillations, which disrupt dendritic spine structure and function, impairing interneuronal communication and contributing to cognitive deficits in AD.^73^

**Figure 3:**
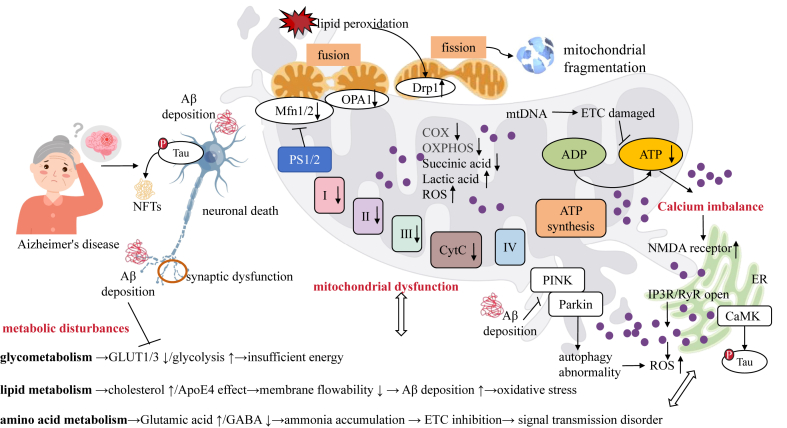
The core pathological network of AD is formed by a triangular cyclic system composed of mitochondrial dysfunction, metabolic disturbances, and calcium homeostasis imbalance, which interact bidirectionally to create a self-reinforcing vicious cycle. Mitochondrial dysfunction manifests as structural damage (swelling, cristae rupture, increased volume/decreased numerical density), energy metabolism abnormalities (reduced COX and Complex I-III activities, elevated lactate/decreased succinate), dynamic imbalance (DRP1 up-regulation/OPA1 down-regulation leading to fragmentation), and autophagy impairment (Aβ-mediated inhibition of PINK1/Parkin), collectively triggering the ATP crisis and ROS accumulation. Metabolic disturbances form a glucose–lipid–amino acid triad of dysregulation: impaired glucose metabolism involves GLUT1/3 suppression and compensatory glycolysis elevation, exacerbating mitochondrial damage through feedback inhibition; lipid abnormalities drive Aβ deposition via cholesterol accumulation, ApoE4 effects, and membrane fluidity reduction; and amino acid imbalance features glutamate/GABA ratio disruption and ammonia toxicity. Calcium dyshomeostasis arises from NMDA receptor/IP3R hyperactivation, inducing a calcium overload that activates proteases, triggers ROS bursts, promotes tau phosphorylation→NFT formation, and collapses the mitochondrial membrane potential. These three systems interact through positive feedback loops—ROS→mtDNA mutations→ETC damage, Aβ→GLUT inhibition→energy crisis, and calcium overload→mitochondrial permeability transition pore opening. ATP depletion and defective autophagy further transform damaged mitochondria into persistent ROS sources, ultimately driving an irreversible neurodegenerative cascade.

Calcium dysregulation in AD poses a critical threat to neuronal survival, disrupting signaling, metabolism, and mitochondrial function. NMDA receptor hyperactivity and excessive ER calcium release via IP3R/RyR channels flood the cytosol with Ca^2+^, overwhelming homeostatic mechanisms.^74^ Elevated cytosolic Ca^2+^ activates CaMK, driving tau hyperphosphorylation.^75^ Hyperphosphorylated tau detaches from microtubules, destabilizing them and forming NFTs, which disrupt neuronal structure and synaptic transmission.^76^ Calcium dysregulation also alters gene expression and protein synthesis, impairing pathways critical for neuronal survival.^77^ Calcium overload triggers apoptosis by activating proteases, phospholipases, and endonucleases, which degrade cytoskeletal proteins, hydrolyze membrane lipids, and fragment DNA.^78^ Concurrent mitochondrial dysfunction exacerbates ROS production, oxidizing lipids, proteins, and nucleic acids while disrupting neurotransmitter synthesis and release, impairing cognition and memory^79^ (Fig. 3).

Calcium dysregulation in AD patients is evidenced by up-regulated NMDA receptor subunits and MAM expansion in post-mortem brains. Unlike transient physiological calcium sparks, AD neurons exhibit pathological calcium waves driven by: (1) ER calcium leakage (via IP3R hyperactivation); (2) Impaired mitochondrial uptake (via MCU overexpression); and (3) Plasma membrane influx (via Aβ-activated channels).^80^^,^^81^ These waves activate calpains and caspases, cleaving tau and mitochondrial proteins—a mechanism supported by human tauopathy data showing calcium-dependent tau cleavage products. Notably, calcium stabilizers like nimodipine improve cognition in some AD subgroups, suggesting that calcium dysregulation is not just a downstream effect but a modifiable driver.

## Mitochondria-metabolism-calcium homeostasis interplay

### Mitochondria-metabolism crosstalk

Mitochondrial dysfunction profoundly impacts metabolic pathways. Impaired respiratory chain complexes disrupt electron transfer, hindering NADH/FADH2 oxidation and suppressing OXPHOS, thereby reducing ATP synthesis.^82^ To compensate, cells activate anaerobic glycolysis, increasing glucose uptake and lactate production.^83^ This metabolic shift not only lowers energy efficiency but also elevates intracellular acidity, impairing enzyme activity and signaling.^83^ Mitochondrial defects also obstruct fatty acid β-oxidation and amino acid metabolism. Fatty acid oxidation requires mitochondrial enzymes and transporters; dysfunction leads to lipid accumulation and metabolic imbalance.^84^ Similarly, mitochondrial failure disrupts amino acid catabolism (e.g., glutamate oxidation), causing toxic metabolite buildup.^85^

Under chronic high-sugar diets or diabetic conditions, elevated blood glucose levels lead to excessive glucose influx into cells, overloading mitochondria and inducing functional damage.^86^ Hyperglycemia triggers excessive mitochondrial ROS production, which oxidizes lipids and proteins in mitochondrial membranes, compromising structure and function.^87^ Lipid metabolism abnormalities, such as hypercholesterolemia and hypertriglyceridemia, cause intracellular lipid accumulation, impairing mitochondrial integrity. Excess cholesterol and triglycerides disrupt membrane fluidity and permeability, hindering respiratory chain complexes and other proteins and thereby exacerbating mitochondrial energy metabolism dysfunction.^88^^,^^89^ Additionally, metabolic byproducts like ketones and uric acid accumulate systemically, further impairing mitochondrial performance.^90^

The AMP-activated protein kinase (AMPK) pathway is central to energy homeostasis. Decreased ATP and increased AMP levels activate AMPK, which phosphorylates targets like acetyl-CoA carboxylase (ACC). Phosphorylated ACC reduces fatty acid synthesis while promoting fatty acid oxidation to boost ATP production.^91^ AMPK also enhances mitochondrial biogenesis and repair by regulating transcription factors such as peroxisome proliferator-activated receptor γ coactivator-1α (PGC-1α).^92^ The Sirtuin 1 (SIRT1) pathway, an NAD^+^-dependent deacetylase, modulates mitochondrial function and metabolism. SIRT1 deacetylates substrates like PGC-1α and forkhead box class O3a (FOXO3a), enhancing their activity to promote mitochondrial biogenesis and metabolic homeostasis.^93^^,^^94^ By regulating metabolic genes, SIRT1 maintains glucose and lipid metabolism balance, safeguarding cellular metabolic stability.^94^

### Mitochondria-calcium homeostasis nexus

Mitochondria possess the ability to take in and release calcium ions, with their uptake mechanism primarily relying on the MCU complex located on the mitochondrial inner membrane.^95^ The MCU complex consists of multiple subunits, including MCU, MCUR1, MICU1, and MICU2.^96^ Under normal physiological conditions, when the intracellular calcium ion concentration increases, calcium ions enter the mitochondrial matrix through the MCU complex along the electrochemical gradient.^97^ The presence of the mitochondrial membrane potential provides the driving force for calcium ion uptake; the higher the mitochondrial membrane potential is, the greater the driving force for calcium ion uptake would be. The uptake of calcium ions by mitochondria is also regulated by MICU1 and MICU2, which act as calcium ion sensors. Under low calcium conditions, they can inhibit the activity of MCU, preventing excessive calcium ion uptake by mitochondria.^98^ The release of calcium ions from mitochondria primarily occurs through the mitochondrial NCX and mPTP. NCX facilitates the reverse exchange of calcium ions in the mitochondrial matrix with sodium ions in the cytoplasm, thereby releasing calcium ions.^8^ mPTP is a non-selective large pore channel. Under pathological conditions such as oxidative stress and calcium overload, mPTP opens, leading to the rapid release of calcium ions, small molecules, and macromolecules from the mitochondrial matrix into the cytoplasm. This process disrupts the mitochondrial membrane potential, induces mitochondrial dysfunction, and may even result in apoptosis.^99^

As a critical calcium reservoir, mitochondria suffer functional impairment during cytosolic calcium overload. Excessive Ca^2+^ activates the mitochondrial permeability transition pore (mPTP), collapsing the membrane potential (ΔΨm), uncoupling the respiratory chain, reducing ATP synthesis, releasing apoptogenic factors (e.g., CytC), and increasing ROS production.^100^ Calcium overload also stimulates mitochondrial calcium-dependent proteases and phospholipases, catalyzing lipid peroxidation and proteolysis, which generate more ROS and damage mitochondrial membranes/enzymes, perpetuating dysfunction.^101^

Disruption of calcium homeostasis can severely impair mitochondrial function. When there is intracellular calcium overload, a large amount of calcium ions entering mitochondria can cause mitochondrial membrane potential depolarization, damaging the normal structure and function of mitochondria.^102^ Calcium overload also activates phosphatases and proteases in mitochondria, leading to the degradation of proteins such as mitochondrial respiratory chain complexes and ATP synthase, which inhibits mitochondrial energy metabolism.^103^ Calcium overload in mitochondria promotes the production of ROS, which further oxidizes lipids and proteins on the mitochondrial membrane, creating a vicious cycle that exacerbates mitochondrial damage.^104^ Studies have shown that in the brains of patients with AD, disruption of calcium homeostasis leads to mitochondrial dysfunction, which in turn affects the energy supply and survival of neuronal cells, serving as one of the important mechanisms of AD pathogenesis.^105^

This close link involves multiple important signaling pathways. The opening of mPTP is associated with various signaling pathways, with cyclosporine A-sensitive protein D (CypD) being a crucial component of mPTP.^106^ When cells are stimulated by oxidative stress, calcium overload, and other factors, the expression and activity of CypD increase, promoting the opening of mPTP. The CaMKⅡ signaling pathway is also involved in the regulation of mitochondrial calcium homeostasis.^107^ CaMKⅡ can be activated by the calcium ion–calmodulin complex, and the activated CaMKⅡ can phosphorylate proteins such as MCU, regulating mitochondrial uptake and release of calcium ions.^108^ In the brains of AD patients, the activity of CaMKⅡ is abnormally elevated, leading to disruption of mitochondrial calcium homeostasis, which in turn affects mitochondrial function and the survival of neuronal cells.^109^

### Metabolism-calcium homeostasis interdependence

Metabolic abnormalities can significantly impact calcium homeostasis. In metabolic diseases such as diabetes, hyperglycemia leads to intracellular glucose metabolic disorders, activating the polyol pathway and PKC pathway.^110^ Activation of the polyol pathway results in the accumulation of sorbitol and fructose within cells, causing an increase in intracellular osmolarity, enhanced cell membrane permeability, and increased calcium ion influx.^111^ Activation of the PKC pathway phosphorylates calcium channels and transporters on the cell membrane, altering their function and leading to an imbalance in calcium ion influx and efflux.^112^ Abnormal lipid metabolism also affects calcium homeostasis.^113^ Hypercholesterolemia and hypertriglyceridemia alter the lipid composition of the cell membrane, impacting the function of calcium channels and transporters, and resulting in abnormal calcium ion transport.^114^^,^^115^ Some lipid metabolites, such as oxidized low-density lipoprotein (ox-LDL), can stimulate cells to produce an inflammatory response and release cytokines, which affect calcium channels and transporters on the cell membrane, leading to the disruption of calcium homeostasis.^116^ Disruption of calcium homeostasis also interferes with metabolic pathways. Intracellular calcium overload activates a series of calcium-dependent enzymes, such as phospholipase A2 (PLA2), PKC, and calcineurin (CaN).^117^ Activation of PLA2 leads to the hydrolysis of cell membrane phospholipids, producing metabolites such as arachidonic acid, which further affect cell metabolism and signaling.^118^ Activation of PKC phosphorylates various metabolism-related enzymes and proteins, such as glycogen synthase and pyruvate dehydrogenase, thereby inhibiting glucose metabolism and energy production.^119^ Activation of CaN dephosphorylates the transcription factor NFAT, allowing it to enter the nucleus and regulate the expression of related genes, affecting cell metabolism and function.^120^ Calcium overload also leads to mitochondrial dysfunction, impacting cellular energy metabolism and further exacerbating metabolic disorders.^121^

In this interactive process, multiple important signaling pathways are involved. The IP3R signaling pathway plays a crucial role in the interaction between metabolism and calcium homeostasis.^122^ When cells are stimulated, phospholipase C (PLC) is activated, hydrolyzing phosphatidylinositol 4,5-bisphosphate (PIP2) to generate phosphatidylinositol 3,4,5-trisphosphate (PIP3) and diacylglycerol (DAG). IP3 binds to IP3R on the ER, causing the release of calcium ions stored in the ER into the cytoplasm, thereby increasing the intracellular calcium ion concentration.^123^ The increased intracellular calcium ion concentration further activates signaling pathways such as PKC, affecting metabolic processes.^124^ The PLC signaling pathway is also involved in the regulation of metabolism and calcium homeostasis. Activation of PLC not only produces IP3 but also generates DAG, which can activate PKC. PKC, through phosphorylation of various substrates, regulates the activity of metabolism-related enzymes and proteins, impacting cell metabolism and calcium homeostasis.^125^

### Integrated tripartite model in AD

The interplay among mitochondria, metabolism, and calcium homeostasis in AD forms a complex pathological network. In the early stages of AD, mitochondrial dysfunction may be one of the initiating factors.^126^ Impaired function of mitochondrial respiratory chain complexes leads to reduced ATP synthesis and inadequate energy supply.^127^ To maintain cellular energy demands, cells activate the anaerobic glycolysis pathway, resulting in abnormal glucose metabolism and increased lactate production.^128^ Mitochondrial dysfunction also increases the production of ROS, increasing oxidative stress.^129^ Oxidative stress damages mitochondrial membranes and mtDNA, further exacerbating mitochondrial dysfunction.^130^

Metabolic disturbances also play a crucial role in this process. Metabolic abnormalities such as hyperglycemia and hyperlipidemia can lead to mitochondrial overload and impair mitochondrial function.^131^ The accumulation of pathological proteins, such as Aβ and tau proteins, interferes with intracellular signaling and metabolic processes, causing disruptions in calcium homeostasis.^132^ Aβ can bind to receptors on the cell membrane, activating calcium channels and increasing calcium ion influx.^133^ Hyperphosphorylation of tau proteins disrupts the cytoskeleton, affecting the function of organelles such as the ER and mitochondria, and leading to disruptions in calcium homeostasis.^134^ Imbalances in calcium homeostasis further exacerbate mitochondrial dysfunction and metabolic disturbances. Intracellular calcium overload activates calcium-dependent enzymes, leading to the degradation of proteins such as mitochondrial respiratory chain complexes and ATP synthase, inhibiting mitochondrial energy metabolism.^135^ Calcium overload also promotes ROS production, enhances oxidative stress, and damages mitochondrial membranes and mtDNA. Imbalances in calcium homeostasis affect the activity of metabolism-related enzymes and proteins, interfering with metabolic pathways and leading to an inadequate energy supply.^136^

**Figure 4:**
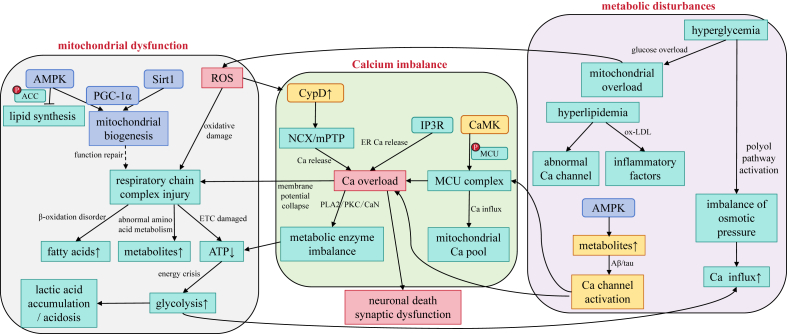
The interplay between mitochondria-metabolism-calcium homeostasis constitutes a core pathological triad in AD: a vicious cycle involving mitochondrial damage (disruption of respiratory chain complexes leading to reduced ATP production, activation of anaerobic glycolysis causing lactic acidosis, and β-oxidation impairment resulting in fatty acid accumulation and lipotoxicity), metabolic storm (hyperglycemia/hyperlipidemia-induced mitochondrial overload and ROS burst, along with Aβ/tau-mediated calcium channel dysfunction), and calcium crisis (MCU-mediated calcium influx combined with pathological mPTP opening, triggering calcium overload, membrane potential collapse, and apoptosis). Compensatory mechanisms partially counteract these damages through the AMPK pathway (energy-sensing inhibition of lipid synthesis via ACC phosphorylation and PGC-1α activation to promote mitochondrial biogenesis) and the SIRT1 axis (NAD^+^-dependent deacetylation enhancing PGC-1α activity for mitochondrial repair). AD-specific pathways include Aβ/tau toxicity (direct activation of calcium channels driving pathological influx), the CypD-mPTP axis (oxidative stress-induced mitochondrial content leakage), and CaMKⅡ dysregulation (aberrant MCU phosphorylation exacerbating calcium uptake imbalance). Cascade amplification manifests as metabolite accumulation (fatty acids/lactate altering membrane fluidity and ion channel function), a ROS-calcium death spiral (ROS↑→mPTP opening→Ca^2+^↑→ROS↑ loop), and inflammatory disruption (ox-LDL-induced ER calcium release via PLC/IP3 signaling). Ultimately, these dysfunctions converge into terminal pathways: CytC release activating apoptotic cascades, PKC/PLA2/CaN-driven metabolic collapse, and hippocampal/cortical neuron loss causing cognitive decline, collectively shaping AD’s irreversible pathological endpoint (red highlights: pathological processes; blue: repair mechanisms; yellow: AD-specific pathways).

The synergistic dysregulation of these three factors collectively promotes the occurrence and progression of AD (Fig. 4). Mitochondrial dysfunction, metabolic disturbances, and disruptions in calcium homeostasis interact, forming a vicious cycle that continuously exacerbates neuronal damage and death.^137^ As the disease progresses, neuronal damage gradually spreads, leading to significant loss of neurons in regions such as the cerebral cortex and hippocampus, ultimately resulting in cognitive and memory dysfunction.^138^

## In-depth analysis of key molecular mechanisms

### The “toxic synergy” of the Aβ and tau proteins

**Figure 5:**
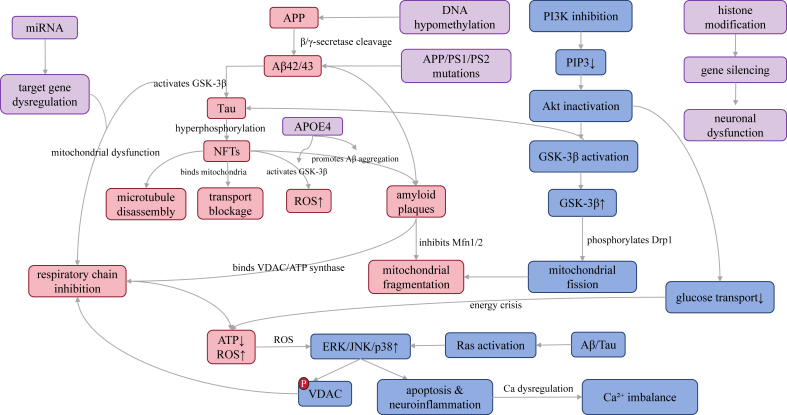
The core pathological mechanism of AD manifests as Aβ-Tau toxic synergy (red module): Aberrant cleavage of APP by β/γ-secretases generates Aβ42/43, which is deposited and directly binds mitochondrial VDAC/ATP synthase to inhibit the respiratory chain, causing ATP depletion and ROS burst. Concurrently, hyperphosphorylated tau forms NFTs, disrupting microtubule structure and blocking mitochondrial transport while activating GSK-3β to amplify ROS production. This creates a self-reinforcing loop—Aβ activates GSK-3β to promote tau phosphorylation, while tau aggregation reciprocally enhances Aβ deposition. In signaling pathway dysregulation (blue module), PI3K-Akt pathway suppression releases GSK-3β (exacerbating tau pathology) and collapses glucose metabolism; MAPK hyperactivation (ERK-mediated VDAC phosphorylation damaging mitochondria, JNK/p38 triggering apoptosis/neuroinflammation) combines with GSK-3β-driven mitochondrial fission via Drp1 phosphorylation. Genetic–epigenetic dysregulation (purple module) involves APP/PS mutations and the APOE4 allele that promotes Aβ overproduction/deposition, DNA hypomethylation/aberrant histone modifications that causes pathological protein accumulation, and miRNA imbalance that worsens mitochondrial gene dysfunction. Through cross-module cascades, ROS bursts activate MAPK pathways to disrupt calcium homeostasis, while PI3K-Akt inhibition-induced energy crisis and epigenetic abnormalities synergistically amplify Aβ/Tau toxicity, ultimately forming an irreversible neurodegenerative pathological network.

The Aβ and tau proteins play central roles in AD, engaging in a lethal “toxic interplay” that drives disease progression, and profoundly impacting mitochondria, metabolism, and calcium homeostasis (Fig. 5). Aβ is a peptide produced through sequential cleavage of APP by β-secretase and γ-secretase, generating isoforms such as Aβ1-40, Aβ1-42, and Aβ1-43.^139^ In AD brains, an imbalance in the Aβ42/43 ratio—marked by increased hydrophobic, aggregation-prone Aβ42/43—leads to senile plaque formation, a hallmark of AD pathology.^140^ Aβ accumulation disrupts mitochondrial function by interacting with membrane proteins like voltage-dependent anion channels (VDACs) and ATP synthase, inhibiting respiration, reducing ATP synthesis, and elevating ROS production.^141^ Aβ also destabilizes mitochondrial dynamics by suppressing the fusion proteins mitofusin 1 and 2 (Mfn1/Mfn2), causing fragmentation and impairing mitochondrial distribution.

Tau, a microtubule-associated protein, stabilizes microtubules under normal conditions, supporting axonal transport. In AD, hyperphosphorylated tau detaches from microtubules, forming NFTs that disrupt neuronal structure and signaling.^142^ Aberrant tau also impairs mitochondrial trafficking by binding to mitochondrial membrane receptors, causing uneven distribution and energy deficits. Additionally, tau activates kinases like GSK-3β, exacerbating mitochondrial ROS generation.^143^

Aβ and tau engage in a vicious cycle: Aβ activates GSK-3β, promoting tau hyperphosphorylation, while phosphorylated tau accelerates Aβ aggregation.^144^ *In vitro* studies have shown that Aβ exposure increases tau aggregation and phosphorylation, and vice versa.^145^ This synergy amplifies mitochondrial dysfunction, metabolic chaos, and calcium dysregulation. Jointly, Aβ and tau reduce glucose uptake, impair glycolysis/OXPHOS, and deplete cellular energy.^146^ They also disrupt calcium homeostasis, triggering Ca^2+^ overload, protease activation, and mitochondrial damage. Mounting evidence highlights Aβ-tau “toxic synergy” as pivotal in AD pathogenesis, driving neuronal death and cognitive decline via mitochondrial/metabolic collapse and calcium toxicity.^147^ Deciphering their interaction mechanisms is critical for unraveling AD pathology and developing targeted therapies.

### Aberrant signaling pathways

In the complex pathological progression of AD, the dysregulation of multiple signaling pathways acts like malfunctioning circuits, disrupting normal physiological signals within cells and severely damaging the finely tuned regulatory networks governing mitochondria, metabolism, and calcium homeostasis. These disruptions serve as critical drivers of disease progression. The PI3K-Akt signaling pathway plays a crucial role in cell growth, survival, and metabolism. However, in AD, this signaling pathway becomes abnormally dysfunctional. Normally, extracellular signaling molecules such as growth factors bind to receptors on the cell membrane, activating PI3K, which catalyzes the conversion of PIP2 to PIP3. PIP3, as a second messenger, recruits Akt to the cell membrane and, with the help of kinases such as phosphatidylinositol-dependent kinase-1 (PDK1) and mammalian target of rapamycin complex 2 (mTORC2), phosphorylates the serine and threonine residues of Akt to activate it.^148^ Activated Akt regulates cellular physiological functions through various pathways, such as inhibiting the activity of GSK-3β to promote cell survival, regulating the expression and activity of glucose transporters to maintain the cellular energy metabolic balance, and activating downstream transcription factors to regulate the expression of related genes.^149^ In the brains of AD patients, the PI3K-Akt signaling pathway is significantly inhibited. Studies have found that Aβ can bind to receptors on the cell membrane, inhibiting the activity of PI3K and reducing the production of PIP3, thereby blocking the activation of Akt.^150^ Abnormal phosphorylation of the tau protein also interferes with the normal conduction of the PI3K-Akt signaling pathway. It can bind to the regulatory subunit of PI3K, inhibiting its activity, and simultaneously activate GSK-3β to further inhibit the activity of Akt.^151^ Inhibition of the PI3K-Akt signaling pathway leads to a series of adverse consequences. It enhances the activity of GSK-3β, promoting abnormal phosphorylation of the tau protein and the formation of NFTs, which disrupts the structure and function of neurons.^151^ It affects the expression and activity of glucose transporters, resulting in reduced glucose uptake and utilization by neurons, abnormal energy metabolism, and inadequate cellular energy supply.^152^ Additionally, it inhibits the activity of downstream transcription factors, affecting the expression of related genes and impairing neuronal survival and function.

The MAPK signaling pathway plays an important regulatory role in cell proliferation, differentiation, apoptosis, and stress responses. However, in AD, this signaling pathway is abnormally activated. The MAPK signaling pathway mainly includes three pathways: extracellular signal-regulated kinase (ERK), c-Jun N-terminal kinase (JNK), and p38 MAPK.^153^ Normally, extracellular signaling molecules such as growth factors, cytokines, and stress stimuli bind to receptors on the cell membrane, triggering a series of cascade reactions that activate Ras protein, which then activates kinases such as RAF and mitogen-activated protein (MEK), ultimately phosphorylating and activating MAPK.^154^ Activated MAPK enters the nucleus, phosphorylates downstream transcription factors, and regulates the expression of related genes, thereby affecting cellular physiological functions.^155^ In the brains of AD patients, pathological products such as the Aβ and tau proteins can activate the MAPK signaling pathway. Aβ can bind to receptors on the cell membrane, activating the Ras-RAF-MEK-ERK pathway and leading to excessive activation of ERK.^156^ Abnormal phosphorylation of the tau protein can also activate the JNK and p38 MAPK pathways, enhancing their activities.^157^ Abnormal activation of the MAPK signaling pathway has negative effects on mitochondria, metabolism, and calcium homeostasis. Overactivated ERK can phosphorylate mitochondrial-associated proteins such as voltage-dependent anion channel (VDAC) and Mitofusin, leading to mitochondrial dysfunction, abnormal energy metabolism, and increased ROS production.^158^ Activation of JNK and p38 MAPK can induce the expression of apoptosis-related genes, promoting neuronal apoptosis, and activate the expression of inflammation-related genes, triggering neuroinflammatory responses and further damaging neurons and mitochondria.^159^ Activation of the MAPK signaling pathway also affects the expression and activity of calcium homeostasis regulatory proteins, resulting in abnormal elevation of the intracellular calcium ion concentration and disruption of calcium homeostasis.

As a serine/threonine kinase, GSK-3β plays an important regulatory role in cell metabolism, proliferation, differentiation, and apoptosis. However, in AD, GSK-3β becomes a key factor disrupting cellular homeostasis. Normally, the activity of GSK-3β is regulated by multiple signaling pathways, such as the PI3K-Akt signaling pathway, which inhibits its activity by phosphorylating the serine residue of GSK-3β.^160^ GSK-3β participates in various physiological processes, such as regulating glycogen synthesis, cell cycle progression, and gene expression.^161^ In the brains of AD patients, the activity of GSK-3β is significantly enhanced. Aβ can activate GSK-3β, phosphorylating the tau protein, leading to abnormal phosphorylation of the tau protein and the formation of NFTs.^162^ Abnormal phosphorylation of tau protein can further activate GSK-3β, creating a vicious cycle.^163^ Enhanced activity of GSK-3β also affects mitochondrial function by phosphorylating mitochondrial-associated proteins such as Drp1, increasing mitochondrial fission, exacerbating fragmentation, and increasing abnormal energy metabolism and ROS production.^164^ GSK-3β also interferes with the activity of metabolism-related enzymes, affecting the metabolism of glucose, lipids, and amino acids, leading to metabolic disorders.^165^ GSK-3β also affects the phosphorylation state of calcium homeostasis regulatory proteins, resulting in abnormal elevation of the intracellular calcium ion concentration and disruption of calcium homeostasis.

### “Regulatory aberrations” in genes and epigenetics

Mutations in APP, PSEN1, and PSEN2 are primary familial AD (FAD) drivers. These disrupt APP processing via β- and γ-secretases, leading to overproduction and aggregation of neurotoxic Aβ42/43 peptides. Aβ aggregates form senile plaques, trigger neuroinflammation, and directly impair mitochondrial function (reducing respiratory chain activity, ATP synthesis, increasing ROS).^166^, ^167^, ^168^, ^169^, ^170^, ^171^ Critically, mutant presenilins (PSEN1/PS2) interact with mitochondrial membrane proteins, exacerbating structural and functional damage. Furthermore, they disrupt ER calcium storage, deplete ER calcium levels, perturb calcium signaling, and contribute to neuronal death.^172^^,^^173^ PSEN1 mutations, the most common type of FAD, often correlate with early onset and rapid progression.^174^ The APOE ε4 allele is the strongest genetic risk factor for sporadic AD (SAD). The APOE4 protein has a high affinity for Aβ, promoting its aggregation into plaques and hindering its clearance.^175^, ^176^, ^177^, ^178^, ^179^ It also exacerbates tau hyperphosphorylation and NFT formation. Crucially, APOE4 compromises mitochondrial respiration, diminishes ATP production, elevates ROS, disrupts brain glucose and lipid metabolism, and exacerbates neuroinflammation.^180^, ^181^, ^182^ These combined effects severely impact mitochondrial-metabolic fitness and calcium buffering capacity.

In addition to APOE, variations in genes directly governing mitochondrial dynamics (MFN2, OPA1, DRP1), mitophagy (PINK1, PARKIN), metabolic pathways (sortilin-related receptor (SORL1), Clusterin), and calcium handling (inositol 1,4,5-trisphosphate receptor type 3 (ITPR3), RyRs) are increasingly linked to AD risk.^44^^,^^183^ For example, impaired mitophagy due to PINK1/PARKIN dysfunction allows damaged mitochondria to accumulate, fueling oxidative stress and metabolic failure. Dysregulation in calcium channel genes can lead to cytosolic calcium overload, triggering mPTP opening and apoptosis.^45^

Epigenetic modifications alter gene expression without changing DNA sequences and are dynamically influenced by aging and environment. Aberrant methylation occurs in AD brains. Hypomethylation of the APP promoter increases APP expression, boosting Aβ production. Hypermethylation of the PSEN1 promoter can suppress γ-secretase activity, also contributing to Aβ pathology.^184^^,^^185^ Importantly, methylation changes in the promoters of mitochondrial biogenesis regulators (e.g., PGC-1α) and metabolic enzymes can impair energy production and antioxidant responses.^186^ Similarly, altered methylation in genes encoding calcium pumps/channels (e.g., SERCA, PMCA) may disrupt intracellular calcium homeostasis. AD involves altered histone marks. Increased H3K9 methylation silences neuroprotective genes, while decreased H4K16 acetylation affects chromatin structure and gene expression.^187^^,^^188^ Crucially, aberrant histone acetylation/methylation can repress the transcription of mitochondrial electron transport chain components, metabolic enzymes (e.g., PDH complex), and calcium buffering proteins (e.g., calbindin), directly linking epigenetic dysregulation to mitochondrial dysfunction, metabolic deficits, and calcium dysregulation.^188^^,^^189^

Dysregulated miRNAs in AD target key mRNAs. For instance, decreased miR-132 elevates the expression of targets involved in tau phosphorylation, mitochondrial fission/fusion, and calcium signaling (e.g., polypyrimidine tract binding protein 1 (PTBP1), Forkhead box O3 (FOXO3a), and methyl-CpG-binding protein 2 (MECP2)), contributing to neurodegeneration and mitochondrial/metabolic/calcium imbalance.^190^ Other miRNAs (e.g., miR-34a targeting SIRT1, miR-338 targeting COXIV) directly impact mitochondrial biogenesis and oxidative phosphorylation.^191^^,^^192^ Altered lncRNA expression (e.g., BACE1-AS, SRY-box transcription factor 2 (SOX2) overlapping transcript (Sox2-OT)) in AD can promote Aβ production, tau pathology, and neuroinflammation. Significantly, specific lncRNAs (e.g., maternally expressed 3 (MEG3), RNA component of mitochondrial RNA-processing endoribonuclease (RMRP)) are implicated in regulating mitochondrial metabolism, ROS production, and calcium-dependent apoptosis pathways, providing another epigenetic layer influencing the mitochondrial-metabolic-calcium axis in AD.^193^^,^^194^

## The “exploration path” of treatment strategies

### The “repair strategy” of mitochondria-targeted therapy

As an emerging strategy in the field of AD treatment, mitochondria-targeted therapy aims to provide new hope to AD patients by repairing mitochondrial function. It mainly includes several aspects such as mitochondrial protectants, mitophagy regulators, and mitochondrial gene therapy. Mitochondrial protectants are a class of drugs that can directly act on mitochondria, enhance their antioxidant capacity, stabilize the membrane potential, and promote energy metabolism, just like putting on a sturdy armor for mitochondria. Coenzyme Q10 (CoQ10), as a natural antioxidant, is an important component of the mitochondrial respiratory chain. It can participate in the electron transport process, promote the synthesis of ATP, and has antioxidant effects, being able to scavenge the ROS produced by mitochondria and protect mitochondria from oxidative damage.^195^ Studies have shown that in AD animal models, the supplementation of CoQ10 can improve mitochondrial function, reduce the aggregation and deposition of Aβ, and enhance cognitive ability.^196^ However, human trials have shown limited efficacy: a 16-month Phase III trial (NCT00117403) found that high-dose CoQ10 (1200 mg/day) failed to significantly slow cognitive decline in mild–moderate AD patients compared to placebo, despite target engagement in cerebro-spinal fluid (CSF) biomarkers.^197^ This translational gap may reflect species-specific mitochondrial vulnerability, late intervention timing, or insufficient targeting of non-oxidative mechanisms. Idebenone is a synthetic ubiquinone analog with strong antioxidant activity. It can penetrate the blood–brain barrier (BBB), enter neurons, and protect mitochondria from oxidative stress damage.^198^ Clinical studies have shown that idebenone can improve the cognitive function and activities of daily living of AD patients and delay the progression of the disease.^199^

Mitophagy activators, by activating the mitophagy pathway, promote the clearance of damaged mitochondria, thus maintaining the quality and function of mitochondria, just like the “garbage cleaners” in the cell are strengthened. The PINK1/Parkin pathway is a key regulatory pathway of mitophagy, and some small-molecule compounds can promote mitophagy by activating this pathway.^199^ Machine learning-based screening of a large library of small molecules has identified kaempferol and emodin as lead compounds that induce significant autophagy in human cells, *Caenorhabditis elegans*, and the mouse nervous systems. These compounds exert marked improvements in neurodegenerative changes in AD mouse models, including the inhibition of key pathological hallmarks such as extracellular Aβ deposits, intracellular Aβ1-42 accumulation, and the aggregation of microtubule-associated proteins, alongside enhanced learning and memory abilities.^200^ Associate Professor Yi Juan from the School of Basic Medicine of Lanzhou University, in collaboration with the team of Professor Shen Hanming from the University of Macau, found that spautin-1 is a specific inhibitor of mitophagy. By targeting the mitochondrial outer membrane translocase complex, it positively regulates the stability and activity of PINK1, a key molecule for the initiation of mitophagy on the mitochondrial outer membrane, and promotes mitophagy activity. It has also been confirmed that spautin-1 can improve the pathological symptoms of AD in the *Caenorhabditis elegans* model by promoting mitophagy.^201^

Mitochondrial gene therapy is an emerging treatment strategy. It imports normal mitochondrial genes into cells to repair or replace damaged mitochondrial genes, thus improving mitochondrial function, just like rewriting the correct program for mitochondria. mtDNA mutations are one of the important causes of mitochondrial dysfunction. Through gene therapy technology, normal mtDNA can be introduced into cells to correct the mutations and restore the normal function of mitochondria.^202^ However, mitochondrial gene therapy still faces many technical challenges at present, such as the selection of gene vectors, the improvement of gene delivery efficiency, and the regulation of gene expression. Nevertheless, with the continuous development and improvement of gene therapy technology, mitochondrial gene therapy is expected to become an effective means for AD treatment. Mitochondria-targeted therapy provides new ideas and methods for the treatment of AD. By repairing mitochondrial function, it is expected to delay the progression of AD and improve the quality of life of patients.^203^ However, currently, these treatment strategies are still in the research stage and require further in-depth research and clinical trials to verify their safety and effectiveness. It is believed that in the future, with the continuous progress of science and technology, mitochondria-targeted therapy will bring new breakthroughs to the treatment of AD.

### The “balancing strategy” of metabolic regulation therapy

As one of the important strategies for AD treatment, metabolic regulation therapy aims to regulate the metabolic balance of substances such as glucose, lipids, and amino acids, improve the energy supply and function of neurons, and thus open up new avenues for the treatment of AD. Glucose metabolism regulation is one of the key links in metabolic regulation therapy, with the goal of improving the abnormal glucose uptake and utilization in the brains of AD patients and providing sufficient energy for neurons. Glucagon-like peptide-1 (GLP-1) analogs, as a new type of therapeutic drug, have shown good application prospects. GLP-1 is a peptide hormone secreted by intestinal endocrine cells, and it can regulate the glucose metabolism in the brain through multiple pathways. The activation of the GLP-1 receptor can interact with various signaling pathways related to energy metabolism, stimulate glucose-dependent insulin secretion, and inhibit gastric emptying, thereby regulating the glucose metabolic pathways in the brain and promoting an improvement in metabolic capacity.^204^^,^^205^ Studies have shown that GLP-1 analogs can improve the cognitive function of AD patients through multiple mechanisms, such as regulating glucose metabolism, the anti-inflammatory response, and synaptic plasticity.^206^ In AD animal models, administering GLP-1 analogs can increase brain glucose uptake, improve energy metabolism, inhibit 3-Hydroxy-3-methylglutaryl-CoA (HMG-CoA) reductase, reduce Aβ deposition, alleviate neuroinflammatory responses, and thus improve cognitive function.^207^ Clinically, epidemiological studies have associated statin use with a reduced AD incidence, but randomized controlled trials (e.g., HPS, PROSPER) have shown no cognitive benefits in elderly populations.^208^ Subgroup analyses even suggested accelerated cognitive decline in APOE4 carriers. This discrepancy may arise from: (1) Compensatory up-regulation of cerebral cholesterol synthesis; (2) Differential blood–brain barrier penetration across statins; and (3) Disruption of neuroprotective isoprenoid pathways.^209^^,^^210^

Lipid metabolism regulation is also an important direction of metabolic regulation therapy. Its core lies in correcting the disorder of lipid metabolism in the brains of AD patients, reducing the accumulation of harmful lipids, and maintaining the normal structure and function of the cell membrane. Some studies have found that statins, as commonly used lipid-lowering drugs, may have potential therapeutic effects on AD. Statins can inhibit the activity of cholesterol synthase and reduce cholesterol levels, thereby reducing the production and aggregation of Aβ.^208^ Statins can also regulate the inflammatory response and improve mitochondrial function, having a positive impact on the pathological process of AD.^211^ Researchers from Saarland University in Germany and SRH University of Applied Health Sciences in Leverkusen found that the production of Aβ protein affects the synthesis of certain fats, especially a class of lipids called sulfatides, and that the quantity of sulfatides, in turn, regulates the quantity of the Aβ protein. This two-way interaction provides a new target for the treatment of AD.^212^ By regulating the metabolism of sulfatides, it may help reduce the accumulation of the Aβ protein and thus relieve the symptoms of AD.

Amino acid metabolism regulation cannot be ignored either. Its purpose is to maintain the balance of amino acid levels in the brains of AD patients, ensure the normal synthesis and metabolism of neurotransmitters, and promote signal transmission between neurons. Some studies have shown that supplementing certain amino acids or regulating the activity of amino acid-metabolizing enzymes may be beneficial for the treatment of AD. Supplementing glutamate can increase the level of glutamate in the brain, enhance the excitability of neurons, and improve cognitive function.^213^ However, excessive glutamate can also lead to excitotoxicity, so the dosage of amino acid supplementation needs to be precisely controlled.^214^ Regulating the activity of amino acid-metabolizing enzymes, such as glutamine synthetase and glutamate decarboxylase, can also affect the synthesis and metabolism of neurotransmitters such as glutamate and GABA, thereby regulating the balance between the excitability and inhibition of neurons.^215^ Metabolic regulation therapy provides a new strategy for the treatment of AD by regulating the metabolic balance of substances such as glucose, lipids, and amino acids. Although these treatment methods are still in the research stage, they have shown potential therapeutic effects and are expected to bring new hope to AD patients. In the future, further in-depth research on the specific mechanisms and optimal treatment regimens of metabolic regulation therapy is needed to improve the safety and effectiveness of treatment.

### The “stabilizing strategy” of calcium homeostasis regulation therapy

Calcium homeostasis regulation therapy plays an important role in the treatment of AD. Its core objective is to regulate the intracellular calcium ion concentration and restore calcium homeostasis, thereby reducing neuronal damage and improving the pathological process of AD. Calcium channel blockers are one of the important drugs in calcium homeostasis regulation therapy. Their mechanism of action is to prevent the influx of extracellular calcium ions, reduce the intracellular calcium ion concentration, and then affect the contraction activities of cardiac muscle and vascular smooth muscle. In the treatment of AD, they mainly regulate the calcium homeostasis of neurons.^216^ There are many commonly used calcium channel blockers in clinical practice, such as verapamil, diltiazem, and dihydropyridine drugs such as nitrendipine, nimodipine, and nisoldipine.^216^ These drugs can selectively block the calcium channels of the cell membrane and reduce the influx of extracellular calcium ions. Taking nimodipine as an example, it has a high selectivity for cerebral blood vessels, can dilate cerebral blood vessels, increase cerebral blood flow, and improve the ischemic and hypoxic phenomena of brain tissue.^217^ In the treatment of AD, nimodipine can reduce the calcium overload of neurons by inhibiting the influx of calcium ions, thereby reducing the production and aggregation of Aβ and protecting neurons from damage.^218^ Studies have shown that in AD animal models, administering nimodipine can improve cognitive function, reduce the formation of NFTs, and delay the progression of the disease.^219^ In contrast, Phase III trials in mild–moderate AD patients (e.g., the Mini-Mental State Examination (MMSE) study) showed no significant improvement in primary endpoints (AD Assessment Scale-cognitive subscale (ADAS-cog), clinicians’ interview-based impression of change-plus (CIBIC-Plus)), though post hoc analyses suggested benefits in subgroups with severe white matter lesions.^220^^,^^221^ Key limitations include: (1) Compensatory calcium influx through non-L-type channels (e.g., NMDA-R); (2) Late-stage intervention after irreversible synaptic loss; and (3) Inability to reverse Aβ/tau-mediated mPTP dysregulation.^222^

**Figure 6:**
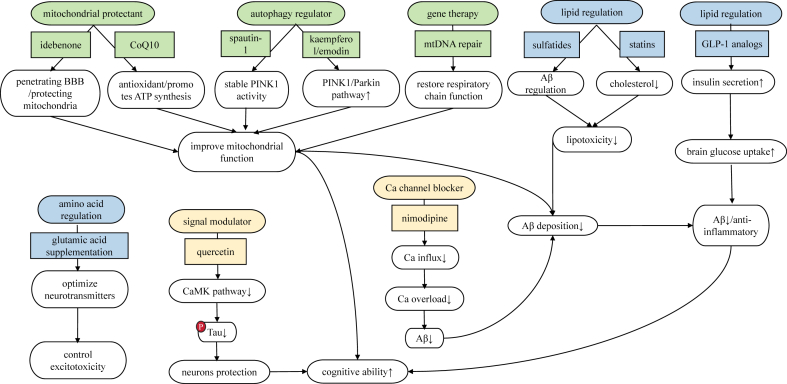
The mitochondrial repair strategy (green) employs protectants like CoQ10 to enhance antioxidant capacity and idebenone to cross the BBB for mitochondrial structural protection. Autophagy activators such as kaempferol/emodin (via the PINK1/Parkin pathway) and spautin-1 (stabilizing PINK1 activity) clear damaged mitochondria, while gene therapy targets mtDNA mutations to restore respiratory chain function. These interventions synergistically improve mitochondrial function, reduce Aβ deposition and enhance cognition. The metabolic balancing strategy (blue) includes glucose regulation (GLP-1 analogs boosting cerebral glucose utilization to suppress Aβ/neuroinflammation), lipid modulation (statins lowering cholesterol and sulfatides bidirectionally regulating Aβ levels), and amino acid optimization (controlled glutamate supplementation to avoid excitotoxicity). Lipid homeostasis reduces the Aβ burden, indirectly shielding mitochondria. The calcium stabilization strategy (orange) uses channel blockers (e.g., nimodipine inhibiting Ca^2+^ influx to alleviate neuronal overload) and signaling modulators (quercetin suppressing the CaMK pathways to reduce tau hyperphosphorylation). Calcium homeostasis maintenance curbs Aβ generation and safeguards mitochondrial function.

Calcium signal regulators maintain calcium homeostasis by regulating the activity of the intracellular calcium signaling pathway. Some natural compounds and small-molecule drugs have shown potential therapeutic effects in this regard. Quercetin, as a natural flavonoid compound, has multiple biological activities, such as antioxidant, anti-inflammatory, and regulation of calcium signals.^223^ Studies have found that quercetin can inhibit the Aβ-induced calcium overload in neurons by regulating the calcium signaling pathway, reduce the production of ROS, and thus protect neurons from oxidative damage.^224^ In cell experiments, quercetin can reduce the intracellular calcium ion concentration in neurons treated with Aβ and alleviate the damage to neurons caused by the imbalance of calcium homeostasis.^223^ Some small-molecule drugs have also been developed to regulate the calcium signaling pathway. They can interact with key proteins in the calcium signaling pathway, regulate the activity and function of the proteins, and thus maintain calcium homeostasis.^225^ These small-molecule drugs can inhibit the excessive activation of signaling pathways such as CaMK, reduce the abnormal phosphorylation of tau protein, and thus improve the pathological symptoms of AD.^226^ Calcium homeostasis regulation therapy provides an effective strategy for the treatment of AD by regulating the intracellular calcium ion concentration and the calcium signaling pathway. Although these treatment methods still need further clinical research to verify their safety and effectiveness, they bring new hope for the treatment of AD (Fig. 6). In the future, with the in-depth study of the mechanism of calcium homeostasis regulation, more effective therapeutic drugs and methods are expected to be developed, bringing new breakthroughs to the treatment of AD patients.

## Conclusions and perspectives

Significant progress has been made in elucidating the molecular mechanisms of mitochondrial-metabolic-calcium homeostasis in AD, providing novel perspectives and a robust theoretical foundation for understanding its pathogenesis. Studies have clearly demonstrated that mitochondrial dysfunction, metabolic disturbances, and calcium dyshomeostasis play pivotal roles in AD progression, forming an intricate pathological network through their interconnected interactions. The abnormal accumulation and interplay of the Aβ and tau proteins, along with dysregulated signaling pathways such as PI3K-Akt, MAPK, and GSK-3β, act as central drivers exacerbating mitochondrial impairment, metabolic dysregulation, and calcium imbalance, ultimately leading to neuronal death and cognitive decline.

However, current research still faces limitations. While numerous AD-associated molecules and pathways have been identified, their precise interactions and regulatory networks remain incompletely understood. Key knowledge gaps persist regarding the molecular mechanisms underlying Aβ/tau aggregation and their specific impacts on mitochondrial dysfunction and calcium dysregulation. Technologically, existing methods for monitoring mitochondrial, metabolic, and calcium homeostasis parameters lack the capacity for comprehensive dynamic analysis of these complex pathophysiological processes. Although traditional cellular and animal models provide essential insights, they exhibit inherent discrepancies compared to human AD pathology, limiting their translational accuracy.

Future breakthroughs in AD research are anticipated across multiple fronts. In fundamental research, emerging technologies such as multi-omics integration, single-cell/spatial transcriptomics, and artificial intelligence (AI)-driven big data analytics will enable deeper exploration of AD mechanisms. Multi-omics integration (genomics, transcriptomics, proteomics, and metabolomics) will facilitate the reconstruction of comprehensive molecular networks, revealing intricate gene-transcript-protein-metabolite relationships. Single-cell and spatial transcriptomics will dissect AD pathogenesis at the cellular and spatial resolution, identifying novel cellular subtypes, biomarkers, and intercellular communication mechanisms. AI-powered data mining will accelerate therapeutic target discovery and precision medicine development.

Therapeutically, mitochondrial-targeted strategies, metabolic modulators, and calcium homeostasis regulators show promising potential but require optimization. Key priorities include developing effective mitochondrial protectants, mitophagy activators, and mitochondrial gene therapies; identifying precise metabolic targets to restore glucose/lipid/amino acid equilibrium; and discovering selective calcium channel modulators. Concurrently, advancing early diagnostic methods and preventive interventions will be crucial for slowing disease progression and improving patient outcomes. Research on mitochondrial-metabolic-calcium homeostasis mechanisms in AD holds tremendous scientific and clinical significance. Through continued innovation and interdisciplinary collaboration, we may ultimately unravel AD pathogenesis and develop transformative therapies, bringing new hope for overcoming this global health challenge and improving the lives of AD patients worldwide.

Despite these advances, critical barriers hinder translation from preclinical data to clinical therapies. The mitochondrial and metabolic dysfunction emerges decades before symptoms, yet most trials intervene at later stages when pathology is irreversible, limiting its impact. Pathological heterogeneity further complicates progress—AD subtypes exhibit distinct triad dysregulation (e.g., mitochondrial-predominant *vs.* calcium-driven dysfunction), making one-size-fits-all treatments ineffective.^227^ Single-target therapies (e.g., MCU inhibitors) may activate bypass pathways like store-operated calcium entry (SOCE).^228^ Mouse models inadequately recapitulate human metabolic aging and APOE4 interactions. Human induced pluripotent stem cell (iPSC)-derived neurons and organoids better model cell-type-specific vulnerabilities but lack systemic metabolism.

Future strategies should prioritize biomarker-guided early intervention, which entails combining PET to assess metabolic activity, mitochondrial ROS sensors to monitor mitochondrial health, and calcium-sensitive functional magnetic resonance imaging (fMRI) to track calcium dynamics—enabling interventions at preclinical stages when pathological changes are still reversible. Additionally, combinatorial approaches that simultaneously target multiple components of the mitochondrial-metabolic-calcium triad, such as pairing mitophagy inducers with lactate transport inhibitors, hold promise for disrupting the self-reinforcing pathological cycles. Equally important is patient stratification based on triad-specific endophenotypes, such as distinguishing between “metabolic AD” and “calcium-associated tauopathy”, to ensure more precise and effective therapeutic targeting.

## CRediT authorship contribution statement

**Tingting Liu:** Writing – review & editing, Writing – original draft. **Zongting Rong:** Conceptualization, Data curation, Writing – review & editing. **Jingwen Li:** Visualization, Software. **Haojie Wu:** Writing – original draft. **Jianshe Wei:** Writing – review & editing, Funding acquisition.

## Funding

This work was supported partly by the 10.13039/501100001809National Natural Science Foundation of China (No. 32161143021, 81271410), the Henan University graduate “Talent Program” of Henan Province, China (No. SYLYC2023092), and the 10.13039/501100006407Henan Natural Science Foundation of China (No. 182300410313).

## Conflict of interests

The authors declare no competing interests.

## References

[1] Scheltens P., De Strooper B., Kivipelto M. (2021). Alzheimer’s disease. Lancet.

[2] Golde T.E. (2022). Alzheimer’s disease - the journey of a healthy brain into organ failure. Mol Neurodegener.

[3] Li X., Wu Z., Si X., Li J., Wu G., Wang M. (2025). The role of mitochondrial dysfunction in the pathogenesis of Alzheimer’s disease and future strategies for targeted therapy. Eur J Med Res.

[4] Sushma Mondal AC. (2019). Role of GPCR signaling and calcium dysregulation in Alzheimer’s disease. Mol Cell Neurosci.

[5] Goleij P., Khazeei Tabari M.A., Poudineh M. (2025). Therapeutic potential of melatonin-induced mitophagy in the pathogenesis of Alzheimer’s disease. Inflammopharmacology.

[6] Morán M., Moreno-Lastres D., Marín-Buera L., Arenas J., Martín M.A., Ugalde C. (2012). Mitochondrial respiratory chain dysfunction: implications in neurodegeneration. Free Radic Biol Med.

[7] Atlante A., de Bari L., Bobba A., Amadoro G. (2017). A disease with a sweet tooth: exploring the Warburg effect in Alzheimer’s disease. Biogerontology.

[8] Ge M., Zhang J., Chen S. (2022). Role of calcium homeostasis in Alzheimer’s disease. Neuropsychiatric Dis Treat.

[9] Calvo-Rodriguez M., Bacskai B.J. (2021). Mitochondria and calcium in Alzheimer’s disease: from cell signaling to neuronal cell death. Trends Neurosci.

[10] Hidalgo-Gutiérrez A., González-García P., Díaz-Casado M.E. (2021). Metabolic targets of coenzyme Q10 in mitochondria. Antioxidants.

[11] Gejl M., Gjedde A., Egefjord L. (2016). In Alzheimer’s disease, 6-month treatment with GLP-1 analog prevents decline of brain glucose metabolism: randomized, placebo-controlled, double-blind clinical trial. Front Aging Neurosci.

[12] Joshi M., Joshi S., Khambete M., Degani M. (2023). Role of calcium dysregulation in Alzheimer’s disease and its therapeutic implications. Chem Biol Drug Des.

[13] Roger A.J., Muñoz-Gómez S.A., Kamikawa R. (2017). The origin and diversification of mitochondria. Curr Biol.

[14] Li W., Long Q., Wu H. (2022). Nuclear localization of mitochondrial TCA cycle enzymes modulates pluripotency *via* histone acetylation. Nat Commun.

[15] Taleva G., Husová M., Panicucci B. (2023). Mitochondrion of the *Trypanosoma brucei* long slender bloodstream form is capable of ATP production by substrate-level phosphorylation. PLoS Pathog.

[16] Martínez-Reyes I., Chandel N.S. (2020). Mitochondrial TCA cycle metabolites control physiology and disease. Nat Commun.

[17] Lunt S.Y., Vander Heiden M.G. (2011). Aerobic glycolysis: meeting the metabolic requirements of cell proliferation. Annu Rev Cell Dev Biol.

[18] Chini C.C.S., Zeidler J.D., Kashyap S., Warner G., Chini E.N. (2021). Evolving concepts in NAD^+^ metabolism. Cell Metab.

[19] Jeon Y.G., Kim Y.Y., Lee G., Kim J.B. (2023). Physiological and pathological roles of lipogenesis. Nat Metab.

[20] Song Y., Wade H., Zhang B. (2023). Polymorphisms of fat mass and obesity-associated gene in the pathogenesis of child and adolescent metabolic syndrome. Nutrients.

[21] Zhang Y., Hu J., Lu P., Yang R., Liang X.F., Liu L. (2024). Addition of α-ketoglutaric acid (AKG) reduces deamination in Chinese perch (*Siniperca chuatsi*) fed with fermented soybean meal as a substitute for fishmeal. Fish Physiol Biochem.

[22] Pitts R.F., Stone W.J. (1967). Renal metabolism of alanine. J Clin Investig.

[23] Habegger K.M. (2022). Cross talk between insulin and glucagon receptor signaling in the hepatocyte. Diabetes.

[24] Berridge M.J., Bootman M.D., Roderick H.L. (2003). Calcium signalling: dynamics, homeostasis and remodelling. Nat Rev Mol Cell Biol.

[25] Ramakrishna S., Jhaveri V., Konings S.C. (2021). APOE4 affects basal and NMDAR-mediated protein synthesis in neurons by perturbing calcium homeostasis. J Neurosci.

[26] Cui C., Merritt R., Fu L., Pan Z. (2017). Targeting calcium signaling in cancer therapy. Acta Pharm Sin B.

[27] Ye L., Zeng Q., Ling M. (2021). Inhibition of IP3R/Ca^2+^ dysregulation protects mice from ventilator-induced lung injury *via* endoplasmic reticulum and mitochondrial pathways. Front Immunol.

[28] Garbincius J.F., Elrod J.W. (2022). Mitochondrial calcium exchange in physiology and disease. Physiol Rev.

[29] Gnegy M.E. (1993). Calmodulin in neurotransmitter and hormone action. Annu Rev Pharmacol Toxicol.

[30] Lamers J.M., Ruigrok T.J. (1983). Diminished Na^+^/K^+^ and Ca^2+^ pump activities in the Ca^2+^ depleted heart: possible role in the development of Ca^2+^ overload during the Ca^2+^ paradox. Eur Heart J.

[31] Qin Z., Zhou X., Gomez-Smith M. (2012). LIM domain only 4 (LMO4) regulates calcium-induced calcium release and synaptic plasticity in the hippocampus. J Neurosci.

[32] Gowda P., Reddy P.H., Kumar S. (2022). Deregulated mitochondrial microRNAs in Alzheimer’s disease: focus on synapse and mitochondria. Ageing Res Rev.

[33] Wang W., Zhao F., Ma X., Perry G., Zhu X. (2020). Mitochondria dysfunction in the pathogenesis of Alzheimer’s disease: recent advances. Mol Neurodegener.

[34] Yang L., Wu C., Li Y. (2022). Long-term exercise pre-training attenuates Alzheimer’s disease-related pathology in a transgenic rat model of Alzheimer’s disease. Geroscience.

[35] Silva D.F., Selfridge J.E., Lu J., Lezi E., Cardoso S.M., Swerdlow R.H. (2012). Mitochondrial abnormalities in Alzheimer’s disease: possible targets for therapeutic intervention. Adv Pharmacol.

[36] Zhang H., Wei W., Zhao M. (2021). Interaction between aβ and tau in the pathogenesis of Alzheimer’s disease. Int J Biol Sci.

[37] Savvatis K., Vissing C.R., Klouvi L. (2022). Cardiac outcomes in adults with mitochondrial diseases. J Am Coll Cardiol.

[38] Trushina E., Trushin S., Hasan M.F. (2022). Mitochondrial complex I as a therapeutic target for Alzheimer’s disease. Acta Pharm Sin B.

[39] Singh A., Kukreti R., Saso L., Kukreti S. (2019). Oxidative stress: a key modulator in neurodegenerative diseases. Molecules.

[40] Wang Q., Duan L., Li X. (2022). Glucose metabolism, neural cell senescence and Alzheimer’s disease. Int J Mol Sci.

[41] Minhas P.S., Jones J.R., Latif-Hernandez A. (2024). Restoring hippocampal glucose metabolism rescues cognition across Alzheimer’s disease pathologies. Science.

[42] Nikolac Perkovic M., Videtic Paska A., Konjevod M. (2021). Epigenetics of Alzheimer’s disease. Biomolecules.

[43] Filadi R., Greotti E., Turacchio G., Luini A., Pozzan T., Pizzo P. (2016). Presenilin 2 modulates endoplasmic reticulum-mitochondria coupling by tuning the antagonistic effect of mitofusin 2. Cell Rep.

[44] Pires M., Rego A.C. (2023). Apoe4 and Alzheimer’s disease pathogenesis-mitochondrial deregulation and targeted therapeutic strategies. Int J Mol Sci.

[45] Zhou T.Y., Ma R.X., Li J. (2023). Review of PINK1-Parkin-mediated mitochondrial autophagy in Alzheimer’s disease. Eur J Pharmacol.

[46] Qiu W.Q., Ai W., Zhu F.D. (2022). *Polygala* saponins inhibit NLRP3 inflammasome-mediated neuroinflammation *via* SHP-2-Mediated mitophagy. Free Radic Biol Med.

[47] Yang C., Pan R.Y., Guan F., Yuan Z. (2024). Lactate metabolism in neurodegenerative diseases. Neural Regen Res.

[48] Sun C., Liu X., Wang B. (2019). Endocytosis-mediated mitochondrial transplantation: transferring normal human astrocytic mitochondria into glioma cells rescues aerobic respiration and enhances radiosensitivity. Theranostics.

[49] Pan R.Y., He L., Zhang J. (2022). Positive feedback regulation of microglial glucose metabolism by histone H4 lysine 12 lactylation in Alzheimer’s disease. Cell Metab.

[50] Hu Y., Zou H., Zhong Z. (2024). The role of astrocyte-neuron lactate shuttle in neuropathic orofacial pain. J Oral Rehabil.

[51] Blázquez E., Hurtado-Carneiro V., LeBaut-Ayuso Y. (2022). Significance of brain glucose hypometabolism, altered insulin signal transduction, and insulin resistance in several neurological diseases. Front Endocrinol.

[52] Wei L., Yang X., Wang J. (2023). H3K18 lactylation of senescent microglia potentiates brain aging and Alzheimer’s disease through the NFκB signaling pathway. J Neuroinflammation.

[53] Yan X., Hu Y., Wang B., Wang S., Zhang X. (2020). Metabolic dysregulation contributes to the progression of Alzheimer’s disease. Front Neurosci.

[54] Mangalmurti A., Lukens J.R. (2022). How neurons die in Alzheimer’s disease: implications for neuroinflammation. Curr Opin Neurobiol.

[55] Dewanjee S., Chakraborty P., Bhattacharya H. (2022). Altered glucose metabolism in Alzheimer’s disease: role of mitochondrial dysfunction and oxidative stress. Free Radic Biol Med.

[56] Wang X., Yang J., Zhang X. (2024). An endophenotype network strategy uncovers YangXue QingNao Wan suppresses Aβ deposition, improves mitochondrial dysfunction and glucose metabolism. Phytomedicine.

[57] Feizabadi M.S., Castillon V.J. (2022). The effect of tau and taxol on polymerization of MCF7 microtubules *in vitro*. Int J Mol Sci.

[58] Dienel G.A. (2019). Brain glucose metabolism: integration of energetics with function. Physiol Rev.

[59] Yin F. (2023). Lipid metabolism and Alzheimer’s disease: clinical evidence, mechanistic link and therapeutic promise. FEBS J.

[60] Kunkle B.W., Grenier-Boley B., Sims R. (2019). Genetic meta-analysis of diagnosed Alzheimer’s disease identifies new risk loci and implicates Aβ, tau, immunity and lipid processing. Nat Genet.

[61] Wroński A., Wójcik P. (2022). Impact of ROS-dependent lipid metabolism on psoriasis pathophysiology. Int J Mol Sci.

[62] Serrano-Pozo A., Das S., Hyman B.T. (2021). APOE and Alzheimer’s disease: advances in genetics, pathophysiology, and therapeutic approaches. Lancet Neurol.

[63] Andersen J.V., Schousboe A., Verkhratsky A. (2022). Astrocyte energy and neurotransmitter metabolism in Alzheimer’s disease: integration of the glutamate/GABA-glutamine cycle. Prog Neurobiol.

[64] Czapski G.A., Strosznajder J.B. (2021). Glutamate and GABA in microglia-neuron cross-talk in Alzheimer’s disease. Int J Mol Sci.

[65] McGill Percy K.C., Liu Z., Qi X. (2025). Mitochondrial dysfunction in Alzheimer’s disease: guiding the path to targeted therapies. Neurotherapeutics.

[66] Kumar V., Kim S.H., Bishayee K. (2022). Dysfunctional glucose metabolism in Alzheimer’s disease onset and potential pharmacological interventions. Int J Mol Sci.

[67] Xu L., Liu R., Qin Y., Wang T. (2023). Brain metabolism in Alzheimer’s disease: biological mechanisms of exercise. Transl Neurodegener.

[68] Yu W., Jin H., Huang Y. (2021). Mitochondria-associated membranes (MAMs): a potential therapeutic target for treating Alzheimer’s disease. Clin Sci (Lond).

[69] Ooi K., Hu L., Feng Y. (2021). Sigma-1 receptor activation suppresses microglia M1 polarization *via* regulating endoplasmic reticulum-mitochondria contact and mitochondrial functions in stress-induced hypertension rats. Mol Neurobiol.

[70] Cao Y., Chen Z., Hu J. (2021). Mfn2 regulates high glucose-induced MAMs dysfunction and apoptosis in podocytes *via* PERK pathway. Front Cell Dev Biol.

[71] Bazargani N., Attwell D. (2016). Astrocyte calcium signaling: the third wave. Nat Neurosci.

[72] Princen K., Van Dooren T., van Gorsel M. (2024). Pharmacological modulation of septins restores calcium homeostasis and is neuroprotective in models of Alzheimer’s disease. Science.

[73] Zhang Z., Yang X., Song Y.Q., Tu J. (2021). Autophagy in Alzheimer’s disease pathogenesis: therapeutic potential and future perspectives. Ageing Res Rev.

[74] Yu T.W., Lane H.Y., Lin C.H. (2021). Novel therapeutic approaches for Alzheimer’s disease: an updated review. Int J Mol Sci.

[75] Wani A., Al Rihani S.B., Sharma A. (2021). Crocetin promotes clearance of amyloid-β by inducing autophagy *via* the STK11/LKB1-mediated AMPK pathway. Autophagy.

[76] Jack CR Jr, Bennett D.A., Blennow K. (2018). NIA-AA research framework: toward a biological definition of Alzheimer’s disease. Alzheimer’s Dement.

[77] Nixon R.A. (2024). Autophagy-lysosomal-associated neuronal death in neurodegenerative disease. Acta Neuropathol.

[78] Kotian V., Sarmah D., Kaur H. (2019). Evolving evidence of calreticulin as a pharmacological target in neurological disorders. ACS Chem Neurosci.

[79] Zhou B., Tian R. (2018). Mitochondrial dysfunction in pathophysiology of heart failure. J Clin Investig.

[80] Jadiya P., Kolmetzky D.W., Tomar D. (2019). Impaired mitochondrial calcium efflux contributes to disease progression in models of Alzheimer’s disease. Nat Commun.

[81] Cascella R., Cecchi C. (2021). Calcium dyshomeostasis in Alzheimer’s disease pathogenesis. Int J Mol Sci.

[82] Wei Y., Du X., Guo H., Han J., Liu M. (2024). Mitochondrial dysfunction and Alzheimer’s disease: pathogenesis of mitochondrial transfer. Front Aging Neurosci.

[83] Sun S., Li H., Chen J., Qian Q. (2017). Lactic acid: No longer an inert and end-product of glycolysis. Physiology.

[84] Bhatti J.S., Bhatti G.K., Reddy P.H. (2017). Mitochondrial dysfunction and oxidative stress in metabolic disorders - a step towards mitochondria based therapeutic strategies. Biochim Biophys Acta Mol Basis Dis.

[85] Prado C.M., Landi F., Chew S.T.H. (2022). Advances in muscle health and nutrition: a toolkit for healthcare professionals. Clin Nutr.

[86] Prasun P. (2020). Mitochondrial dysfunction in metabolic syndrome. Biochim Biophys Acta Mol Basis Dis.

[87] Rovira-Llopis S., Bañuls C., Diaz-Morales N., Hernandez-Mijares A., Rocha M., Victor V.M. (2017). Mitochondrial dynamics in type 2 diabetes: pathophysiological implications. Redox Biol.

[88] Hou Y., Tan E., Shi H. (2024). Mitochondrial oxidative damage reprograms lipid metabolism of renal tubular epithelial cells in the diabetic kidney. Cell Mol Life Sci.

[89] Sun Y., Ge X., Li X. (2020). High-fat diet promotes renal injury by inducing oxidative stress and mitochondrial dysfunction. Cell Death Dis.

[90] Cheng H.L., Chang W.T., Lin J.L. (2023). Mei-gin formula ameliorates obesity through lipolysis, fatty oxidation, and thermogenesis in high-fat diet-induced obese rats. Foods.

[91] Hu Y., Tian C., Chen F., Zhang A., Wang W. (2024). The mystery of methylmercury-perturbed calcium homeostasis: AMPK-DRP1-dependent mitochondrial fission initiates ER-mitochondria contact formation. Sci Total Environ.

[92] Fontecha-Barriuso M., Martin-Sanchez D., Martinez-Moreno J.M. (2020). The role of PGC-1α and mitochondrial biogenesis in kidney diseases. Biomolecules.

[93] Zeng Y., He Y., Wang L. (2024). Dihydroquercetin improves experimental acute liver failure by targeting ferroptosis and mitochondria-mediated apoptosis through the SIRT1/p53 axis. Phytomedicine.

[94] Xie W., Zhu T., Zhang S., Sun X. (2022). Protective effects of Gypenoside XVII against cerebral ischemia/reperfusion injury *via* SIRT1-FOXO3A- and Hif1a-BNIP3-mediated mitochondrial autophagy. J Transl Med.

[95] D’Angelo D., Rizzuto R. (2023). The mitochondrial calcium uniporter (MCU): molecular identity and role in human diseases. Biomolecules.

[96] Patron M., Checchetto V., Raffaello A. (2014). MICU1 and MICU2 finely tune the mitochondrial Ca^2+^ uniporter by exerting opposite effects on MCU activity. Mol Cell.

[97] Prasad Panda S., Kesharwani A. (2023). Micronutrients/miRs/ATP networking in mitochondria: clinical intervention with ferroptosis, cuproptosis, and calcium burden. Mitochondrion.

[98] Stevens T.L., Cohen H.M., Garbincius J.F., Elrod J.W. (2024). Mitochondrial calcium uniporter channel gatekeeping in cardiovascular disease. Nat Cardiovasc Res.

[99] Korotkov S.M. (2023). Mitochondrial oxidative stress is the general reason for apoptosis induced by different-valence heavy metals in cells and mitochondria. Int J Mol Sci.

[100] He P., Liu F., Li M. (2023). Mitochondrial calcium ion nanogluttons alleviate periodontitis *via* controlling mPTPs. Adv Healthcare Mater.

[101] Raimondi M., Fontana F., Marzagalli M. (2021). Ca^2+^ overload- and ROS-associated mitochondrial dysfunction contributes to δ-tocotrienol-mediated paraptosis in melanoma cells. Apoptosis.

[102] Csordás G., Weaver D., Hajnóczky G. (2018). Endoplasmic reticulum-mitochondrial contactology: structure and signaling functions. Trends Cell Biol.

[103] Zhang Y.Y., Yang X.Y., Liu H.Q. (2023). The weakened interaction between HECTD4 and GluN2B in ischemic stroke promotes calcium overload and brain injury through a mechanism involving the decrease of GluN2B and MALT1 ubiquitination. Mol Neurobiol.

[104] Lin Y., Jiang M., Chen W., Zhao T., Wei Y. (2019). Cancer and ER stress: Mutual crosstalk between autophagy, oxidative stress and inflammatory response. Biomed Pharmacother.

[105] Panes-Fernandez J., Godoy P.A., Gavilan J. (2023). TG2 promotes amyloid beta aggregates: impact on ER-mitochondria crosstalk, calcium homeostasis and synaptic function in Alzheimer’s disease. Biomed Pharmacother.

[106] Robichaux D.J., Harata M., Murphy E., Karch J. (2023). Mitochondrial permeability transition pore-dependent necrosis. J Mol Cell Cardiol.

[107] Gordan R., Fefelova N., Gwathmey J.K., Xie L.H. (2020). Iron overload, oxidative stress and calcium mishandling in cardiomyocytes: role of the mitochondrial permeability transition pore. Antioxidants.

[108] LaMoia T.E., Hubbard B.T., Guerra M.T. (2024). Cytosolic calcium regulates hepatic mitochondrial oxidation, intrahepatic lipolysis, and gluconeogenesis *via* CAMKII activation. Cell Metab.

[109] Lee A., Kondapalli C., Virga D.M. (2022). Aβ42 oligomers trigger synaptic loss through CAMKK2-AMPK-dependent effectors coordinating mitochondrial fission and mitophagy. Nat Commun.

[110] Bhattamisra S.K., Koh H.M., Lim S.Y., Choudhury H., Pandey M. (2021). Molecular and biochemical pathways of catalpol in alleviating diabetes mellitus and its complications. Biomolecules.

[111] Tang W.H., Cheng W.T., Kravtsov G.M. (2010). Cardiac contractile dysfunction during acute hyperglycemia due to impairment of SERCA by polyol pathway-mediated oxidative stress. Am J Physiol Cell Physiol.

[112] Kuravi S.J., Ahmed N.S., Taylor K.A. (2023). Delineating zinc influx mechanisms during platelet activation. Int J Mol Sci.

[113] Zhao J., He C., Fan X. (2024). Tripeptidyl peptidase II coordinates the homeostasis of calcium and lipids in the central nervous system and its depletion causes presenile dementia in female mice through calcium/lipid dyshomeostasis-induced autophagic degradation of CYP19A1. Theranostics.

[114] Abrams J. (2005). Clinical practice. Chronic stable angina. N Engl J Med.

[115] Pal S., Ghosh M., Ghosh S., Bhattacharyya S., Sil P.C. (2015). Atorvastatin induced hepatic oxidative stress and apoptotic damage *via* MAPKs, mitochondria, calpain and caspase12 dependent pathways. Food Chem Toxicol.

[116] Li Z., Li J., Li L. (2024). Klotho enhances stability of chronic kidney disease atherosclerotic plaques by inhibiting GRK2/PLC-β-mediated endoplasmic reticulum stress in macrophages *via* modulation of the ROS/SHP1 pathway. Sci Rep.

[117] Gürsoy M., Büyükuysal R.L. (2010). Mechanism of S100b release from rat cortical slices determined under basal and stimulated conditions. Neurochem Res.

[118] Santiago Valtierra F.X., Urriola-Muñoz P., Godoy-Sepúlveda R. (2024). Nonylphenol releases arachidonic acid in rat Sertoli cells *via* activation of PKA and PLA2. Reproduction.

[119] Zhang C., Gu L., Xie H. (2024). Glucose transport, transporters and metabolism in diabetic retinopathy. Biochim Biophys Acta Mol Basis Dis.

[120] Mackiewicz J., Lisek M., Boczek T. (2023). Targeting CaN/NFAT in Alzheimer’s brain degeneration. Front Immunol.

[121] Fefelova N., Wongjaikam S., Pamarthi S.H. (2023). Deficiency of mitochondrial calcium uniporter abrogates iron overload-induced cardiac dysfunction by reducing ferroptosis. Basic Res Cardiol.

[122] Zhang T., Chen S., Li L. (2024). PFKFB3 controls acinar IP3R-mediated Ca^2+^ overload to regulate acute pancreatitis severity. JCI Insight.

[123] Cheng Z., Gan W., Xiang Q. (2025). Impaired degradation of PLCG1 by chaperone-mediated autophagy promotes cellular senescence and intervertebral disc degeneration. Autophagy.

[124] Chen L., Shi D., Guo M. (2021). The roles of PKC-δ and PKC-ε in myocardial ischemia/reperfusion injury. Pharmacol Res.

[125] Zhong T., Zhang W., Guo H. (2022). The regulatory and modulatory roles of TRP family channels in malignant tumors and relevant therapeutic strategies. Acta Pharm Sin B.

[126] Ashleigh T., Swerdlow R.H., Beal M.F. (2023). The role of mitochondrial dysfunction in Alzheimer’s disease pathogenesis. Alzheimer’s Dement.

[127] Patro S., Ratna S., Yamamoto H.A. (2021). ATP synthase and mitochondrial bioenergetics dysfunction in Alzheimer’s disease. Int J Mol Sci.

[128] Rabinowitz J.D., Enerbäck S. (2020). Lactate: the ugly duckling of energy metabolism. Nat Metab.

[129] Zorov D.B., Juhaszova M., Sollott S.J. (2014). Mitochondrial reactive oxygen species (ROS) and ROS-induced ROS release. Physiol Rev.

[130] Tan W.J.T., Song L. (2023). Role of mitochondrial dysfunction and oxidative stress in sensorineural hearing loss. Hear Res.

[131] Pinti M.V., Fink G.K., Hathaway Q.A., Durr A.J., Kunovac A., Hollander J.M. (2019). Mitochondrial dysfunction in type 2 diabetes mellitus: an organ-based analysis. Am J Physiol Endocrinol Metab.

[132] Wang L., Yin Y.L., Liu X.Z. (2020). Current understanding of metal ions in the pathogenesis of Alzheimer’s disease. Transl Neurodegener.

[133] Boopathi S., Garduño-Juárez R. (2022). Calcium inhibits penetration of Alzheimer’s Aβ_1_-_42_ monomers into the membrane. Proteins.

[134] Kabir M.T., Sufian M.A., Uddin M.S. (2019). NMDA receptor antagonists: repositioning of memantine as a multitargeting agent for Alzheimer’s therapy. Curr Pharm Des.

[135] Li J., Ma Y., Qiu T. (2024). Autophagy-dependent lysosomal calcium overload and the ATP5B-regulated lysosomes-mitochondria calcium transmission induce liver insulin resistance under perfluorooctane sulfonate exposure. Ecotoxicol Environ Saf.

[136] Huang J., He J., Wang J. (2023). Calcium carbonate-actuated ion homeostasis perturbator for oxidative damage-augmented Ca^2+^/Mg^2+^ interference therapy. Biomaterials.

[137] Xiong J., Kang S.S., Wang Z. (2022). FSH blockade improves cognition in mice with Alzheimer’s disease. Nature.

[138] Yang A.C., Vest R.T., Kern F. (2022). A human brain vascular atlas reveals diverse mediators of Alzheimer’s risk. Nature.

[139] Iulita M.F., Bejanin A., Vilaplana E. (2023). Association of biological sex with clinical outcomes and biomarkers of Alzheimer’s disease in adults with Down syndrome. Brain Commun.

[140] Watanabe H., Yoshida C., Hidaka M., Ogawa T., Tomita T., Futai E. (2022). Specific mutations in Aph1 cause γ-secretase activation. Int J Mol Sci.

[141] Battogtokh G., Choi Y.S., Kang D.S. (2018). Mitochondria-targeting drug conjugates for cytotoxic, anti-oxidizing and sensing purposes: current strategies and future perspectives. Acta Pharm Sin B.

[142] Namme J.N., Bepari A.K., Takebayashi H. (2021). Cofilin signaling in the CNS physiology and neurodegeneration. Int J Mol Sci.

[143] Nisha Sarkar S. (2021). Downregulation of glob1 suppresses pathogenesis of human neuronal tauopathies in *Drosophila* by regulating tau phosphorylation and ROS generation. Neurochem Int.

[144] Wu J.J., Yang Y., Wan Y. (2022). New insights into the role and mechanisms of ginsenoside Rg1 in the management of Alzheimer’s disease. Biomed Pharmacother.

[145] Liu M., Dexheimer T., Sui D. (2020). Hyperphosphorylated tau aggregation and cytotoxicity modulators screen identified prescription drugs linked to Alzheimer’s disease and cognitive functions. Sci Rep.

[146] Miao J., Chen L., Pan X., Li L., Zhao B., Lan J. (2023). Microglial metabolic reprogramming: emerging insights and therapeutic strategies in neurodegenerative diseases. Cell Mol Neurobiol.

[147] Wang Z.J., Li X.R., Chai S.F. (2023). Semaglutide ameliorates cognition and glucose metabolism dysfunction in the 3xTg mouse model of Alzheimer’s disease *via* the GLP-1R/SIRT1/GLUT4 pathway. Neuropharmacology.

[148] Kumar M., Bansal N. (2022). Implications of phosphoinositide 3-kinase-Akt (PI3K-Akt) pathway in the pathogenesis of Alzheimer’s disease. Mol Neurobiol.

[149] Lin J., Song T., Li C., Mao W. (2020). GSK-3β in DNA repair, apoptosis, and resistance of chemotherapy, radiotherapy of cancer. Biochim Biophys Acta Mol Cell Res.

[150] Zhang D., Hu X., Qian L. (2011). Microglial MAC1 receptor and PI3K are essential in mediating β-amyloid peptide-induced microglial activation and subsequent neurotoxicity. J Neuroinflammation.

[151] Liu S., Xu L., Shen Y., Wang L., Lai X., Hu H. (2024). Qingxin Kaiqiao Fang decreases Tau hyperphosphorylation in Alzheimer’s disease *via* the PI3K/Akt/GSK3β pathway *in vitro* and *in vivo*. J Ethnopharmacol.

[152] Butterfield D.A., Halliwell B. (2019). Oxidative stress, dysfunctional glucose metabolism and Alzheimer disease. Nat Rev Neurosci.

[153] Wang L., Lu Y., Liu J. (2024). Gegen Qinlian tablets delay Alzheimer’s disease progression *via* inhibiting glial neuroinflammation and remodeling gut microbiota homeostasis. Phytomedicine.

[154] Hendrikse C.E., Theelen P.M., van der Ploeg P. (2023). The potential of RAS/RAF/MEK/ERK (MAPK) signaling pathway inhibitors in ovarian cancer: a systematic review and meta-analysis. Gynecol Oncol.

[155] Thakur S., Dhapola R., Sarma P., Medhi B., Reddy D.H. (2023). Neuroinflammation in Alzheimer’s disease: current progress in molecular signaling and therapeutics. Inflammation.

[156] Chen Y., Jiang L., Li M., Shen Y., Liu S., Yang D. (2024). Huanglian Jiedu decoction alleviates neurobehavioral damage in mice with chronic alcohol exposure through the RAS-RAF-MEK-ERK pathway. Heliyon.

[157] Hwang J.W., Kim J., Park J.H. (2024). Felodipine attenuates neuroinflammatory responses and tau hyperphosphorylation through JNK/P38 signaling in tau-overexpressing AD mice. Mol Brain.

[158] Dodson M., Darley-Usmar V., Zhang J. (2013). Cellular metabolic and autophagic pathways: traffic control by redox signaling. Free Radic Biol Med.

[159] Xue M.T., Sheng W.J., Song X. (2022). Atractylenolide III ameliorates spinal cord injury in rats by modulating microglial/macrophage polarization. CNS Neurosci Ther.

[160] Wang S., Sudan R., Peng V. (2022). TREM2 drives microglia response to amyloid-β *via* SYK-dependent and-independent pathways. Cell.

[161] Jaworski T. (2020). Control of neuronal excitability by GSK-3beta: epilepsy and beyond. Biochim Biophys Acta Mol Cell Res.

[162] Jiang M., Zhang X., Yan X. (2021). GSK3β is involved in promoting Alzheimer’s disease pathologies following chronic systemic exposure to *Porphyromonas gingivalis* lipopolysaccharide in amyloid precursor protein NL-F/NL-F knock-in mice. Brain Behav Immun.

[163] Lauretti E., Dincer O., Praticò D. (2020). Glycogen synthase kinase-3 signaling in Alzheimer’s disease. Biochim Biophys Acta Mol Cell Res.

[164] Su L., Zhang J., Gomez H., Kellum J.A., Peng Z. (2023). Mitochondria ROS and mitophagy in acute kidney injury. Autophagy.

[165] Zhang S., Lachance B.B., Mattson M.P., Jia X. (2021). Glucose metabolic crosstalk and regulation in brain function and diseases. Prog Neurobiol.

[166] Cai H., Pang Y., Fu X., Ren Z., Jia L. (2023). Plasma biomarkers predict Alzheimer’s disease before clinical onset in Chinese cohorts. Nat Commun.

[167] Hur J.Y. (2022). γ-secretase in Alzheimer’s disease. Exp Mol Med.

[168] Zoltowska K.M., Berezovska O. (2018). Dynamic nature of presenilin1/γ-secretase: implication for Alzheimer’s disease pathogenesis. Mol Neurobiol.

[169] Johri A. (2021). Disentangling mitochondria in Alzheimer’s disease. Int J Mol Sci.

[170] Sehar U., Rawat P., Reddy A.P., Kopel J., Reddy P.H. (2022). Amyloid beta in aging and Alzheimer’s disease. Int J Mol Sci.

[171] Yonemura Y., Futai E., Yagishita S., Kaether C., Ishiura S. (2016). Specific combinations of presenilins and Aph1s affect the substrate specificity and activity of γ-secretase. Biochem Biophys Res Commun.

[172] Park M.W., Cha H.W., Kim J. (2021). NOX4 promotes ferroptosis of astrocytes by oxidative stress-induced lipid peroxidation *via* the impairment of mitochondrial metabolism in Alzheimer’s diseases. Redox Biol.

[173] Kumar M., Haghighi K., Kranias E.G., Sadayappan S. (2020). Phosphorylation of cardiac myosin-binding protein-C contributes to calcium homeostasis. J Biol Chem.

[174] Oakley H., Cole S.L., Logan S. (2006). Intraneuronal beta-amyloid aggregates, neurodegeneration, and neuron loss in transgenic mice with five familial Alzheimer’s disease mutations: potential factors in amyloid plaque formation. J Neurosci.

[175] Xu X., Zhang B., Wang X. (2021). A meta-analysis of Alzheimer’s disease’s relationship with human ApoE gene variants. Am J Transl Res.

[176] Jackson R.J., Meltzer J.C., Nguyen H. (2022). APOE4 derived from astrocytes leads to blood-brain barrier impairment. Brain.

[177] Raulin A.C., Doss S.V., Trottier Z.A., Ikezu T.C., Bu G., Liu C.C. (2022). ApoE in Alzheimer’s disease: pathophysiology and therapeutic strategies. Mol Neurodegener.

[178] Xia Z., Prescott E.E., Urbanek A. (2024). Co-aggregation with Apolipoprotein E modulates the function of Amyloid-β in Alzheimer’s disease. Nat Commun.

[179] Abu-Elfotuh K., Hamdan A.M.E., Mohamed S.A. (2024). The potential anti-Alzheimer’s activity of *Oxalis corniculata* Linn. Methanolic extract in experimental rats: role of APOE4/LRP1, TLR4/NF-κβ/NLRP3, Wnt 3/β-catenin/GSK-3β, autophagy and apoptotic cues. J Ethnopharmacol.

[180] Liu X.T., Chen X., Zhao N., Geng F., Zhu M.M., Ren Q.G. (2024). Synergism of ApoE4 and systemic infectious burden is mediated by the APOE-NLRP3 axis in Alzheimer’s disease. Psychiatr Clin Neurosci.

[181] Frieden C., Wang H., Ho C.M.W. (2017). A mechanism for lipid binding to apoE and the role of intrinsically disordered regions coupled to domain-domain interactions. Proc Natl Acad Sci U S A.

[182] Parhizkar S., Holtzman D.M. (2022). APOE mediated neuroinflammation and neurodegeneration in Alzheimer’s disease. Semin Immunol.

[183] Chen S., Li Q., Shi H., Li F., Duan Y., Guo Q. (2024). New insights into the role of mitochondrial dynamics in oxidative stress-induced diseases. Biomed Pharmacother.

[184] Wei X., Zhang L., Zeng Y. (2020). DNA methylation in Alzheimer’s disease: in brain and peripheral blood. Mech Ageing Dev.

[185] Shi Q., Chowdhury S., Ma R. (2017). Complement C3 deficiency protects against neurodegeneration in aged plaque-rich APP/PS1 mice. Sci Transl Med.

[186] Yokoyama A.S., Rutledge J.C., Medici V. (2017). DNA methylation alterations in Alzheimer’s disease. Environ Epigenet.

[187] De Plano L.M., Saitta A., Oddo S., Caccamo A. (2024). Epigenetic changes in Alzheimer’s disease: DNA methylation and histone modification. Cells.

[188] Qin H.Y., Liu J.Y., Fang C.L., Deng Y.P., Zhang Y. (2023). DNA methylation: the epigenetic mechanism of Alzheimer’s disease. Ibrain.

[189] Xu D.C., Sas-Nowosielska H., Donahue G. (2024). Histone acetylation in an Alzheimer’s disease cell model promotes homeostatic amyloid-reducing pathways. Acta Neuropathol Commun.

[190] Li Y.B., Fu Q., Guo M., Du Y., Chen Y., Cheng Y. (2024). microRNAs: pioneering regulators in Alzheimer’s disease pathogenesis, diagnosis, and therapy. Transl Psychiatry.

[191] Wang L., Sun M., Cao Y. (2020). miR-34a regulates lipid metabolism by targeting SIRT1 in non-alcoholic fatty liver disease with iron overload. Arch Biochem Biophys.

[192] Aschrafi A., Schwechter A.D., Mameza M.G., Natera-Naranjo O., Gioio A.E., Kaplan B.B. (2008). microRNA-338 regulates local cytochrome c oxidase IV mRNA levels and oxidative phosphorylation in the axons of sympathetic neurons. J Neurosci.

[193] Pichet Binette A., Gaiteri C., Wennström M. (2024). Proteomic changes in Alzheimer’s disease associated with progressive Aβ plaque and tau tangle pathologies. Nat Neurosci.

[194] Chen Y., Yu Y. (2023). Tau and neuroinflammation in Alzheimer’s disease: interplay mechanisms and clinical translation. J Neuroinflammation.

[195] Mantle D., Hargreaves I.P. (2022). Mitochondrial dysfunction and neurodegenerative disorders: role of nutritional supplementation. Int J Mol Sci.

[196] Asadbegi M., Komaki H., Faraji N. (2023). Effectiveness of coenzyme Q10 on learning and memory and synaptic plasticity impairment in an aged Aβ-induced rat model of Alzheimer’s disease: a behavioral, biochemical, and electrophysiological study. Psychopharmacology (Berl).

[197] Mantle D., Heaton R.A., Hargreaves I.P. (2021). Coenzyme Q10, ageing and the nervous system: an overview. Antioxidants.

[198] Wang H., Li L., Jia K. (2020). Idebenone protects mitochondrial function against amyloid beta toxicity in primary cultured cortical neurons. Neuroreport.

[199] Zhang J., Zhang Y., Wang J., Xia Y., Zhang J., Chen L. (2024). Recent advances in Alzheimer’s disease: mechanisms, clinical trials and new drug development strategies. Signal Transduct Target Ther.

[200] Xie C., Zhuang X.X., Niu Z. (2022). Amelioration of Alzheimer’s disease pathology by mitophagy inducers identified *via* machine learning and a cross-species workflow. Nat Biomed Eng.

[201] Yi J., Wang H.L., Lu G. (2024). Spautin-1 promotes PINK1-PRKN-dependent mitophagy and improves associative learning capability in an Alzheimer disease animal model. Autophagy.

[202] Hu Z., Yang L., Zhang M. (2024). A novel protein *CYTB*-187AA encoded by the mitochondrial gene *CYTB* modulates mammalian early development. Cell Metab.

[203] Mi Y., Qi G., Brinton R.D., Yin F. (2021). Mitochondria-targeted therapeutics for Alzheimer’s disease: the good, the bad, the potential. Antioxidants Redox Signal.

[204] Nowell J., Blunt E., Gupta D., Edison P. (2023). Antidiabetic agents as a novel treatment for Alzheimer’s and Parkinson’s disease. Ageing Res Rev.

[205] Kopp K.O., Glotfelty E.J., Li Y., Greig N.H. (2022). Glucagon-like peptide-1 (GLP-1) receptor agonists and neuroinflammation: implications for neurodegenerative disease treatment. Pharmacol Res.

[206] Zheng J., Xie Y., Ren L. (2021). GLP-1 improves the supportive ability of astrocytes to neurons by promoting aerobic glycolysis in Alzheimer’s disease. Mol Metabol.

[207] Olmastroni E., Molari G., De Beni N. (2022). Statin use and risk of dementia or Alzheimer’s disease: a systematic review and meta-analysis of observational studies. Eur J Prev Cardiol.

[208] Ye Z., Deng J., Wu X. (2025). Association of statins use and genetic susceptibility with incidence of Alzheimer’s disease. J Prev Alzheimers Dis.

[209] Montagne A., Nation D.A., Sagare A.P. (2020). APOE4 leads to blood-brain barrier dysfunction predicting cognitive decline. Nature.

[210] Narasimhan S., Holtzman D.M., Apostolova L.G. (2024). Apolipoprotein E in Alzheimer’s disease trajectories and the next-generation clinical care pathway. Nat Neurosci.

[211] Petek B., Häbel H., Xu H. (2023). Statins and cognitive decline in patients with Alzheimer’s and mixed dementia: a longitudinal registry-based cohort study. Alzheimers Res Ther.

[212] Zimmer V.C., Lauer A.A., Haupenthal V. (2024). A bidirectional link between sulfatide and Alzheimer’s disease. Cell Chem Biol.

[213] Yang S., Xie Z., Pei T. (2022). Salidroside attenuates neuronal ferroptosis by activating the Nrf2/HO1 signaling pathway in Aβ_1-42_-induced Alzheimer’s disease mice and glutamate-injured HT22 cells. Chin Med.

[214] Peng B., Yang Q., Joshi R.B. (2020). Role of alcohol drinking in Alzheimer’s disease, Parkinson’s disease, and amyotrophic lateral sclerosis. Int J Mol Sci.

[215] Arab H.H., Eid A.H., Yahia R. (2023). Targeting autophagy, apoptosis, and SIRT1/Nrf2 axis with topiramate underlies its neuroprotective effect against cadmium-evoked cognitive deficits in rats. Pharmaceuticals.

[216] Park J.H., Hwang J.W., Lee H.J. (2023). Lomerizine inhibits LPS-mediated neuroinflammation and tau hyperphosphorylation by modulating NLRP3, DYRK1A, and GSK3α/β. Front Immunol.

[217] Danta C.C. (2020). Calcium channel blockers: a possible potential therapeutic strategy for the treatment of Alzheimer’s dementia patients with SARS-CoV-2 infection. ACS Chem Neurosci.

[218] Hashioka S., Wu Z., Klegeris A. (2021). *Glia*-driven neuroinflammation and systemic inflammation in Alzheimer’s disease. Curr Neuropharmacol.

[219] Tollefson G.D. (1990). Short-term effects of the calcium channel blocker nimodipine (Bay-e-9736) in the management of primary degenerative dementia. Biol Psychiatry.

[220] Dubois B., López-Arrieta J., Lipschitz S. (2023). Correction: masitinib for mild-to-moderate Alzheimer’s disease: results from a randomized, placebo-controlled, phase 3, clinical trial. Alzheimers Res Ther.

[221] Nakamura Y., Usui M., Nishikawa T. (2012). CIBIC plus-J assessment using a videotaped method in Alzheimer’s disease patients. Dement Geriatr Cogn Dis Extra.

[222] O’Day D.H. (2023). Alzheimer’s disease beyond calcium dysregulation: the complex interplay between calmodulin, calmodulin-binding proteins and amyloid beta from disease onset through progression. Curr Issues Mol Biol.

[223] Cui Z., Zhao X., Amevor F.K. (2022). Therapeutic application of quercetin in aging-related diseases: SIRT1 as a potential mechanism. Front Immunol.

[224] Xu Y., Zhang J., Li X. (2024). Erjingwan and Alzheimer’s disease: research based on network pharmacology and experimental confirmation. Front Pharmacol.

[225] Gan L., Jiang Q., Huang D. (2025). A natural small molecule alleviates liver fibrosis by targeting apolipoprotein L2. Nat Chem Biol.

[226] Hwang H.Y., Shim J.S., Kim D., Kwon H.J. (2021). Antidepressant drug sertraline modulates AMPK-MTOR signaling-mediated autophagy *via* targeting mitochondrial VDAC1 protein. Autophagy.

[227] Lam B., Masellis M., Freedman M., Stuss D.T., Black S.E. (2013). Clinical, imaging, and pathological heterogeneity of the Alzheimer’s disease syndrome. Alzheimers Res Ther.

[228] Calvo-Rodriguez M., Hernando-Pérez E., López-Vázquez S., Núñez J., Villalobos C., Núñez L. (2020). Remodeling of intracellular Ca^2+^ homeostasis in rat hippocampal neurons aged *in vitro*. Int J Mol Sci.

